# Blimp1^+^ cells generate functional mouse sebaceous gland organoids in vitro

**DOI:** 10.1038/s41467-019-10261-6

**Published:** 2019-05-28

**Authors:** Alona Feldman, Dzmitry Mukha, Itzhak I. Maor, Egor Sedov, Elle Koren, Yahav Yosefzon, Tomer Shlomi, Yaron Fuchs

**Affiliations:** 1 Laboratory of Stem Cell Biology and Regenerative Medicine, Department of Biology, Technion Israel Institute of Technology, 3200003 Haifa, Israel; 2 Lorry Lokey Interdisciplinary Center for Life Sciences & Engineering, Technion Israel Institute of Technology, 3200003 Haifa, Israel; 3 Technion Integrated Cancer Center, Technion Israel Institute of Technology, 3200003 Haifa, Israel; 4 Department of Computer Science, Technion Israel Institute of Technology, 3200003 Haifa, Israel

**Keywords:** Organogenesis, Skin stem cells

## Abstract

Most studies on the skin focus primarily on the hair follicle and interfollicular epidermis, whereas little is known regarding the homeostasis of the sebaceous gland (SG). The SG has been proposed to be replenished by different pools of hair follicle stem cells and cells that resides in the SG base, marked by Blimp1. Here, we demonstrate that single Blimp1^+^ cells isolated from mice have the potential to generate SG organoids in vitro. Mimicking SG homeostasis, the outer layer of these organoids is composed of proliferating cells that migrate inward, undergo terminal differentiation and generating lipid-filled sebocytes. Performing confocal microscopy and mass-spectrometry, we report that these organoids exhibit known markers and a lipidomic profile similar to SGs in vivo. Furthermore, we identify a role for c-Myc in sebocyte proliferation and differentiation, and determine that SG organoids can serve as a platform for studying initial stages of acne vulgaris, making this a useful platform to identify potential therapeutic targets.

## Abstract (web summary)

The sebaceous gland (SG) has been proposed to be replenished by pools of cells, including a population in the SG base, marked by Blimp1. Here, the authors show that Blimp1^+^ cells can establish an organoid model of the SG, which is regulated by c-Myc and can recapitulate the early stages of acne vulgaris.

## Introduction

The sebaceous gland (SG) is a holocrine-secreting appendage within the epidermis that functions as a crucial component of the pilosebaceous unit^1^. In contrast to the cyclic nature of the hair follicle (HF), the SG undergoes continuous replenishment during which proliferating cells situated at the basal layer move inward to generate lipid-filled sebocytes^1^. As differentiated sebocytes mature, they gradually accumulate lipids and are driven toward the necrotic zone of the SG, where they erupt and release their sebum^1^. Interestingly, the lipid content of the sebum is species specific and in humans changes significantly at different ages^2^.

Although SGs play a central role in skin homeostasis, little is known regarding the mechanisms regulating their maintenance, and how their dysregulation may contribute to certain skin pathologies. One of the most widespread dermatological conditions, acne vulgaris, involves multiple factors including dysregulation of the SGs^2^. The pathogenesis of this disease includes hyperactivation of SGs, excessive keratin deposition and an increase in SG size, which is dependent upon sebocyte hyperproliferation and sebum production. This serves as an ideal lipid-rich environment for *Cutibacterium acnes* (*C. acnes*; formerly *P. acnes*) to flourish, which in turn triggers inflammation via the induction of pro-inflammatory cytokines^2^. Notably, SGs can give rise to additional pathologies including SG carcinomas, which can metastasize and result in high mortality^1^. Thus, there is a great need for generating an ex vivo model for specifically studying and manipulating the SG in a three-dimensional (3D) setting.

The SG has been proposed to be replenished by distinct pools of stem cells or progenitors located in the HF or base of the SG^3–7^. A cellular population that has been reported to govern SG homeostasis in mice is defined by the expression of B-lymphocyte-induced nuclear maturation protein 1 (Blimp1)^3^. Blimp1^+^ cells reside at the base of the SG and have been reported to function as a sebocyte progenitor population^3^. Alternatively, it has recently been suggested that Blimp1 functions in terminally differentiated sebocytes^8^ as well as in granular IFE cells^9, 10^. These reports prompted us to examine whether Blimp1^+^ cells cultured in the proper conditions have the capacity to replenish, as well as potentially generate, SG in vitro.

Here, we show that Blimp1^+^ cells isolated from adult mice have the potential to generate SG organoids that recapitulate the features of SGs in vivo. Utilizing this platform, we find that c-Myc regulates both sebocyte proliferation and differentiation as seen in normal SGs. Furthermore, using these organoids we are able to generate a model that recapitulates aspects of acne vulgaris, demonstrate the effects of currently available acne treatments as well as elucidate c-Myc as a potential therapeutic target. Taken together, here we describe a system that can be employed to study the biology of the SG along with a drug screening platform for SG pathologies.

## Results

### Generation of SG-like structures from mouse Blimp1^+^ cells

**Fig. 1:**
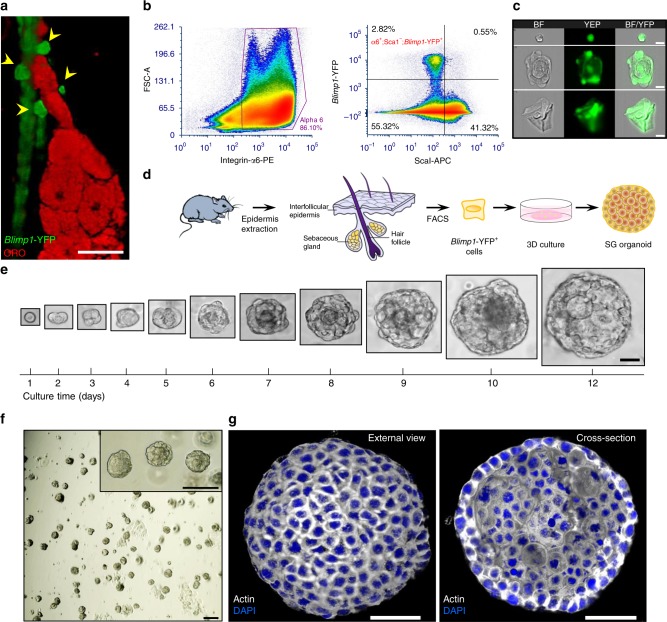
Single Blimp1^+^ cells generate sebaceous gland-like structures ex vivo. **a** Representative image of dorsal skin wholemount extracted from P56 (telogenic)-old *B6.Cg-Tg(Prdm1-EYFP)1Mnz/J* (denoted *Blimp1-YFP*) mouse stained for Oil Red O (ORO). *Blimp1*-YFP^+^ signal (yellow arrows) can be seen at the base of the sebaceous gland (SG) [*n* *=* 5 mice]. **b** Fluorescent-activated cell sorting (FACS) analysis employing *Blimp1-YFP* reporter mice demonstrating the α6^+^;Sca1^-^;*Blimp1*^-^YFP^+^ (*Blimp1*-YFP^+^) population [*n* = 5 pooled mice]. **c** Image stream analysis showing representative brightfield (BF) images of *Blimp1*-YFP^+^ cells, indicating that these cells display three distinct morphologies: (1) small cells with high circularity, (2) large cells resembling sebocytes, and (3) differentiated keratinocytes. **d** Schematic diagram representing the process of *Blimp1*-YFP^+^ cell-derived organoid generation. **e** Representative images of initial single seeded *Blimp1*-YFP^+^ cell maintained in 3D culture for a period of 12 days. Scale bar is relative to all images shown for each day. **f** Single-cell suspension of *Blimp1*-YFP^+^ cells, supplemented with growth factors, form a large number of organoids after a period of 1 week. **g** Representative 3D-reconstructed confocal image of an organoid after 10 days in culture stained for actin. Left panel shows an external view of a layer of compact similarly sized cells, while the right panel demonstrates cross-section of large inner cells of varying sizes. All experiments were repeated at least three times with similar results. Organoids were grown from a mix of at least three mice and cultured in three independent biological repeats. Scale bars: 10 μm (**c**), 20 μm (**a, e**), 50 μm (**g**), 100 μm (**f, f** inset)

We initially examined the distribution of Blimp1^+^ cells in the mouse skin by employing *B6.Cg-Tg(Prdm1-EYFP)1Mnz/J* reporter mice (denoted *Blimp1-YFP*)^11^. Performing confocal Z-stack analyses, we discovered that Blimp1 is expressed in cells found at the base of the SG, as well as in differentiated SG cells and interfollicular epidermal (IFE) granular cells (Fig. 1a and Supplementary Fig. 1a). These findings suggest that Blimp1 does not mark a distinct cell population but is expressed in SG progenitors, mature sebocytes, and differentiated IFE cells, as recently reported^3, 8–10^.

Next, we performed fluorescence-activated cell sorting (FACS) employing *Blimp1-YFP* reporter mice and antibodies against integrin α6 (epidermal keratinocytes) and ScaI (IFE and infundibulum cells). Thereby, α6^+^;ScaI^−^;*Blimp1*-YFP^+^ cells were isolated from 8-week-old mice skin in second telogen (Fig. 1b). Additionally, we could examine the morphology of *Blimp1*-YFP^+^ cells by performing flow cytometry coupled with high-resolution microscopy (ImageStream), verifying that these cells highly express YFP (Fig. 1c and Supplementary Fig. 1b). Furthermore, using this approach, and in accordance with our previous findings, we detected three morphologically distinct groups: (I) small cells with high circularity, (II) large cells resembling mature sebocytes, and (III) differentiated IFE keratinocytes (Supplementary Fig. 1b). Isolated α6^+^;ScaI^−^;*Blimp1*-YFP^+^ cells were then expanded on J2 feeder cells for several passages in 2D culture (Supplementary Fig. 1c), ensuring post-mitotic/terminally differentiated SG/IFE cells were completely excluded. Next, we examined whether embedding cells in a basement membrane-like extracellular 3D environment such as Matrigel, which has been successfully utilized in several epithelial stem cell platforms, could enable establishment of SG organoids (Fig. 1d)^12–14^. Additionally, we supplemented 3D Matrigel cultures with a custom-made media for epidermal cells^15^. In particular, since epithelial cells are extremely sensitive to calcium, bovine serum was chelated and cells were grown in 50 mM of Ca^2+^. Given that several key signaling cascades are known to regulate the function and expansion of epithelial cells in the pilosebaceous unit we also supplemented our culture media with EGF, hFGF-2, Noggin, and R-spondin1 (EFNR). Specifically, we used hFGF-2 as it can drive sebocyte proliferation and hypertrophy^16^, EGF was administered as it enhances sebocyte proliferation, enlarges SGs and increases sebum production^17^ as well as R-spondin1, which promotes Wnt signaling that drives SG generation^18^. We also administered the BMP inhibitor Noggin1 as it has been reported to regulate sebocyte differentiation^19^.

Within one day, we could clearly detect that isolated α6^+^;ScaI^−^;*Blimp1*-YFP^+^ cells had undergone cellular division (Fig. 1e). Strikingly, within 12 days, seeded single cells could generate large spheroid structures, which highly resembled the morphology of a SG in vivo (Fig. 1e). In contrast, when cells were grown without EFNR factors in 3D culture, growth was significantly impaired and only a very limited number of organoids were generated (Supplementary Fig. 1d, e). Of note, a large number of organoids could be generated at a high efficiency (~32% of plated cells) even after multiple passaging (~40 passages) in vitro (Fig. 1f).

Examining the morphology of developed (10-day old) organoids exposed a defined ring of compact cells at the outer layer, as well as larger cells of varying sizes in the inner mass that appeared reminiscent of differentiated sebocytes (Fig. 1g and Supplementary Fig. 1f, Supplementary Movie 1). Notably, when α6^+^;CD34^+^ HFSCs were grown in similar conditions we did not observe the formation of these structures. These cultures resulted in non-organized spheroid clusters highly similar to those recently reported^20^ (Supplementary Fig. 1g). Additionally, under these condition α6^+^;ScaI^+^ IFE/infundibulum cells did not give rise to any defined cellular structure (Supplementary Fig. 1h).

We next examined if we could generate Blimp1^+^ cell-derived organoids by isolating primary α6^+^;ScaI^−^;*Blimp1*-YFP^+^ cells and directly seeding them into Matrigel without a pre-culturing phase. These attempts did not give rise to organoid structures presumably due to low cell survival through loss of cell–cell contact. Thus, we supplemented these cultures with the ROCK inhibitor (Y27632), which prevents cell–cell interaction-dependent anoikis^21^. Under these culture conditions freshly isolated α6^+^;ScaI^−^;*Blimp1*-YFP^+^ cells were able to give rise to organoids, albeit at a lower efficiency (Supplementary Fig. 1i, j).

### SG organoids recapitulate SG features

**Fig. 2:**
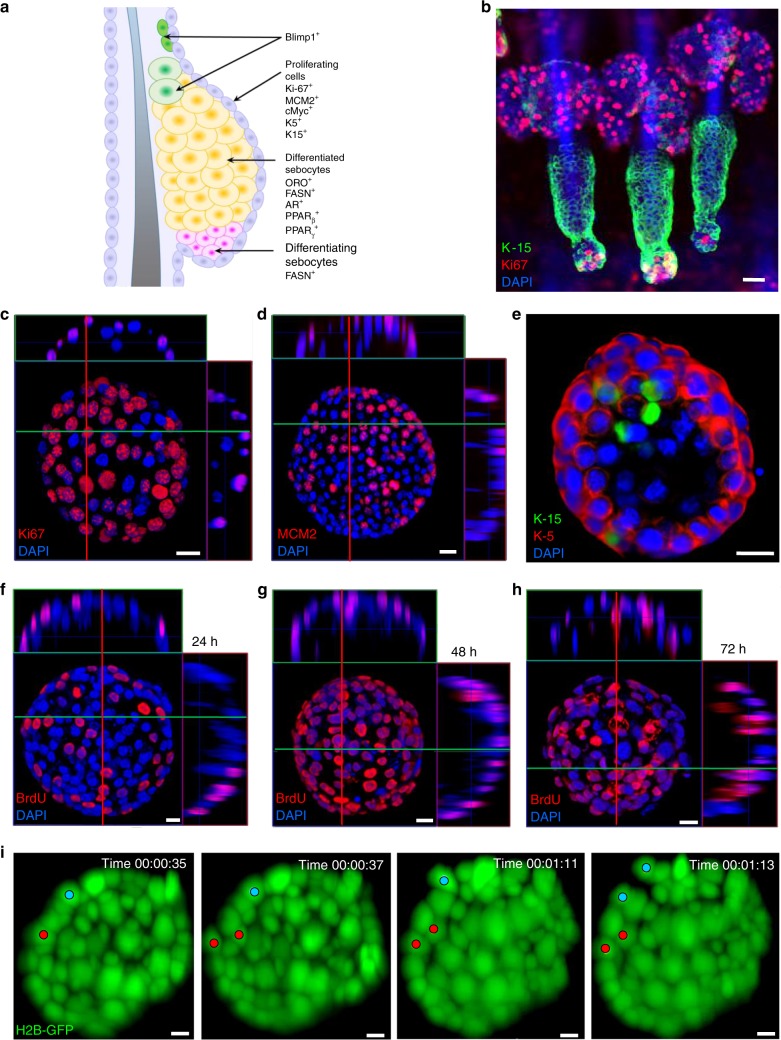
*Blimp1*-YFP^+^ cell-derived organoids express sebaceous gland markers. **a** Schematic view of the sebaceous gland structure and various cell type markers. **b** Tail skin wholemount extracted from P56 (telogenic)-old wild type (WT) mouse stained for Keratin-15 (K-15) and Ki67. **c**, **d** Confocal analysis of *Blimp1*-YFP^+^ generated organoids cultured for 10 days, stained for (**c**) Ki67 and (**d**) MCM2, showing proliferating cells in the outer layer. **e** Confocal analysis of *Blimp1*-YFP^+^-generated organoids cultured for 10 days, stained for K-15 and K-5. **f**–**h** BrdU pulse-chase analysis for (**f**) 24 h, (**g**) 48 h, and (**h**) 72 h showing migration of BrdU-labeled cells from the outer layer to the inner non-proliferating mass. **i** Representative time points of live imaging of movement kinetics, utilizing light sheet fluorescence microscopy, examining 7-day-old organoids derived from *Blimp1*-YFP-H2B-GFP^+^ (nuclear labeled) cells over a 24 h period. Two tracked cells and their progeny (blue and red dots) over time are marked. All images are representative of at least *n* = 3 mice. In vitro experiments were repeated at least three times with similar results. Organoids were grown from a mix of at least three mice and cultured in three independent biological repeats. Scale bars: 10 μm (**i**), 20 μm (**b**–**e**), 50 μm (**f**–**h**)

In the SG, cell proliferation is restricted to basal cells, which reside at a defined region known as the SG proliferative zone (SGPZ). During SG homeostasis, SGPZ cells migrate inward, become post-mitotic and differentiate to lipid-filled sebocytes (Fig. 2a)^1, 22^.

In order to examine whether these organoid structures recapitulate normal SGs, we first performed immunofluorescence (IF) with the proliferative marker, Ki67, known to mark cells of the SGPZ^1, 23^. As expected, analysis of skin wholemounts showed that Ki67 is specifically expressed in the outer layers of the SG and could rarely be seen in quiescent bulge HFSCs in vivo (Fig. 2b). Quantitative analysis indicated that ~34% of cells in the SGPZ were positive for Ki67. Similarly, Ki67^+^ cells could be detected in the outer layer of Blimp1^+^ cell-derived organoids at a similar frequency (Fig. 2c and Supplementary Fig. 2a). These results were also verified using the MCM2-proliferative marker (Fig. 2d and Supplementary Fig. 2b). Notably, alike in vivo, we could not detect proliferating cells within the inner mass of the organoids (Supplementary Fig. 2c, d).

A key component of the cytoskeleton of epithelial cells is Keratin 5 (K-5), which is primarily expressed in basal keratinocytes of the epidermis^24^. Similar to SGs in vivo, Blimp1^+^ cell-derived organoids expressed K-5 in the outer layer (Fig. 2e and Supplementary Fig. 3a, b). Performing IF in both dorsal and tail wholemounts with K-15 antibody, we found that it labels specific cells at the base of the SG and junctional zone, as well as individual cells in the outer layer of the organoids (Fig. 2e and Supplementary Fig. 3a, c).

Similar to the in vivo expression pattern of Blimp1, marking cells at the base of the SG as well as mature sebocytes (Fig. 1a and Supplementary Fig. 1a–c), *Blimp1*-YFP^+^ cells could be seen in both locations in the mature organoid (Supplementary Fig. 3d, e). These findings indicate that the *Blimp1* promoter is active in organoids, supplying further evidence for the similarity to natural SGs.

Since proliferating cells could only be seen on the outer layer of organoids, we investigated whether they could give rise to cells in the inner compartment by monitoring movement kinetics. Conducting pulse-chase 5-bromo-2′-deoxyuridine (BrdU) experiments, we found that 24 h after the pulse only cells located on the organoid outer layer were positive for BrdU (Fig. 2f and Supplementary Fig. 4a). This finding is in accordance with our Ki67 and MCM2 staining (Fig. 2c, d). In contrast, after 48 and 72 h we could clearly detect BrdU^+^ cells in the inner non-proliferating mass, indicating that cells from the outer layer either migrated or proliferated asymmetrically and gave rise to differentiated post-mitotic cells (Fig. 2g, h and Supplementary Fig. 4b, c).

In order to investigate the movement kinetics in real time, we performed time lapse imaging using light sheet microscopy. First, *Blimp1-*YFP^+^ cells were infected with retroviruses encoding H2B-GFP, which fluorescently labels the nucleus. Organoids were then grown for 7 days, followed by visualization for 24 h. Our results indicate that the majority of cellular divisions, estimated at above 90%, occurred along the peripheral ring. However, we could also clearly detect asymmetrical divisions, although at a markedly lower occurrence (Fig. 2i and Supplementary Fig. 4d–f; Supplementary Movie 2). Thus, our data indicate that Blimp1^+^ cell-derived organoids mimic both the expression profile and homeostatic kinetics of SGs in vivo.

### SG organoids display the SG in vivo lipidomic profile

**Fig. 3:**
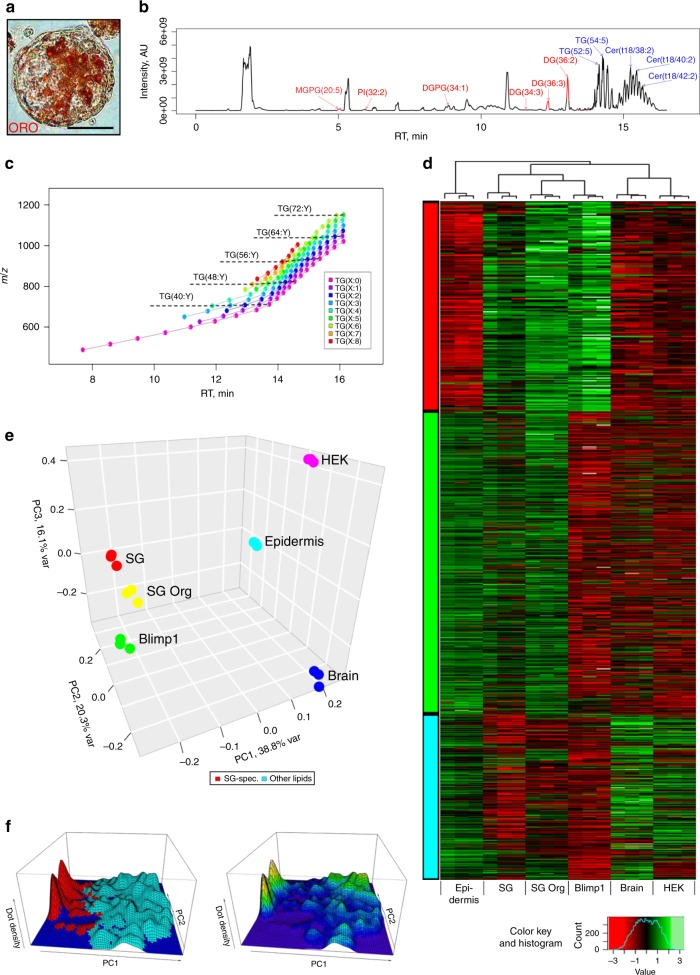
Lipidomic analysis of *Blimp1*-YFP^+^ cell-derived organoids. **a** *Blimp1*-YFP^+^-derived organoid grown for 14 days. Lipids are stained with Oil red O (ORO). **b** Sebaceous gland (SG) sample base peak chromatogram. Lipids specifically abundant in the three SG-like samples: (1) extracted SGs (denoted SG), (2) Blimp1^+^ cell-derived organoids (SG Org), and (3) 2D-grown Blimp1^+^ cells (Blimp1) are marked in red [*n* = 3 independent biological samples]. Several most abundant lipids are marked blue. **c** Representative mz/rt map for triacylglycerides (TGs) peak positions across all superimposed LC/MS samples. Each dot represents the center of a peak group for an identified TG compound. Arrows join peaks separated by mass difference of C_2_H_4_ fragment (5 ppm mass precision) sharing the same number of double bonds. **d** Normalized intensities of lipid compounds identified in the samples. Eighteen columns are arranged by unsupervised hierarchical clustering, while rows are composed according to *k*-means clustering outcome (three clusters: SG/SG-Org/Blimp (cluster I; red), epidermis/SG/SG-Org (cluster II; green), and epidermis/HEK293T cells (HEK)/brain (cluster III; blue). For each sample type, three biological replicates were analyzed. **e** PCA loadings plot shows three major directions identified among samples accounting for 75% of total variance. The main effect is associated with SG features, where loadings are close between SG, SG Org, and Blimp1 samples. The second strongest effect is epidermis-like profile, where loadings are close between epidermis and SG features. **f** Density plot of PCA scores shows separation of total lipidome into two major groups of compounds: SG-specific and the rest of the lipids, as highlighted by color. Density of scores shows non-continuous distribution supporting distinct lipidome profiles for the denoted groups of samples. Scale bar: 50 μm (**a**)

One distinct feature of a SG is the distinct profile of lipids that it generates^25–27^. Using the lipid dye Oil red O (ORO), we found that Blimp^+^ cell-derived organoids encompass lipid-producing cells in the inner mass (Fig. 3a). The observations that inner organoid cells do not proliferate and express lipids is suggestive of a differentiation program similar to that of a SG in vivo. Therefore, in order to examine whether these organoids generate comparable lipids to a normal SG, we performed lipidomic analysis utilizing high-performance liquid chromatography (HPLC) coupled with mass-spectrometry (LC/MS). We performed a comparison of lipid composition between: (1) whole epidermis, (2) resected SGs, (3) Blimp1^+^ cell-derived organoids, and (4) Blimp1^+^ cells grown in 2D culture. Additionally, we included highly unrelated samples: HEK293T cells and mouse brain. Lipids were extracted in three biological replicates by Folch procedure^28^ and were subjected to C18 chromatographic separation, coupled with electrospray ionization high-resolution MS analysis (Fig. 3b). We identified 6470 peaks representing single-charged molecular ions in both polarity modes ([M + H]^+^, [M + NH_4_]^+^, [M-H]^−^, [M + HCO_2_]^−^). Large classes of lipids were observed as series of peaks separated by the mass (*m*/*z*) of C_2_H_4_ segment with a shift to longer retention times (RT) for longer acyl chains and higher saturation levels (Fig. 3c). We used normalized intensity profiles of identified lipid compounds to quantify content similarity between samples. Next, similarity in the total lipidome was assessed by hierarchical clustering (Fig. 3d) and principle component analysis (PCA; Fig. 3e). Hierarchical clustering grouped together resected SGs, Blimp1^+^ cell-derived organoid samples and 2D-grown Blimp1^+^ cells, while HEK293T cells/brain and epidermis represented two additional distinct groups (Fig. 3d, e). A significant portion of detected lipids could then be grouped based on their elevated relative abundance within a combination of sample types: SGs, Blimp1^+^ cell-derived organoids and 2D-grown Blimp1^+^ cells (cluster I), epidermis, SGs, and Blimp1^+^ cell-derived organoids (cluster II) and epidermis, HEK293T cells and brain (cluster III; Fig. 3d). Of note, cluster III was clearly distinguishable from the SGs/organoids/Blimp1^+^ cells (cluster I) profile (Fig. 3d). In our analysis we could detect the production of a specific spectrum of lipids in both normal SGs and in Blimp1^+^ cell-derived organoids (Fig. 3d).

The PCA analysis of the MS data further supported lipidome characterization, by placing the first principle component (PC1) as a direction differentiating SGs, Blimp1^+^ cell-derived organoids and 2D-grown Blimp1^+^ cells from the epidermis and HEK293T cells/brain group of samples (Fig. 3e). The second principle component (PC2) differentiated samples of brain/HEK versus epidermis and SGs/organoids/Blimp1^+^ samples. The first two principle components together explain more than half of the variance in the data. On the PCA score plot, SGs/organoids/Blimp1^+^-specific compounds are distinctly separate from the groups of epidermis and HEK293T cells/brain-specific compounds (Fig. 3e). The density distribution of PCA scores is represented by a landscape with deep valleys separating the SGs/organoids/Blimp1^+^-specific peaks versus all others, indicating the distinction and the strong cross correlation of the SG-like lipids (Fig. 3f). Taken together, our lipidomic analysis indicates that SG organoids have the capacity to generate specific lipids, displaying a signature resembling that of SGs in vivo.

### c-Myc functions as a key regulator of SG organoids

Given that Blimp1^+^ cell-derived organoids displayed morphological and functional similarity to normal SGs, we next examined if this platform could be utilized to investigate signaling cascades that regulate SG homeostasis. One pivotal factor that governs SG expansion is c-Myc^3, 29, 30^. In an in vivo setting, c-Myc labels cells in the SGPZ and regulates both sebocyte proliferation and differentiation^31^ (Supplementary Fig. 5a). Alike normal SGs, we found that the expression of c-Myc mostly co-localized with Ki67 and MCM2 in the SGPZ and, as expected, could not be detected in the inner compartment of the SG organoids (Supplementary Fig. 5b, c).

**Fig. 4:**
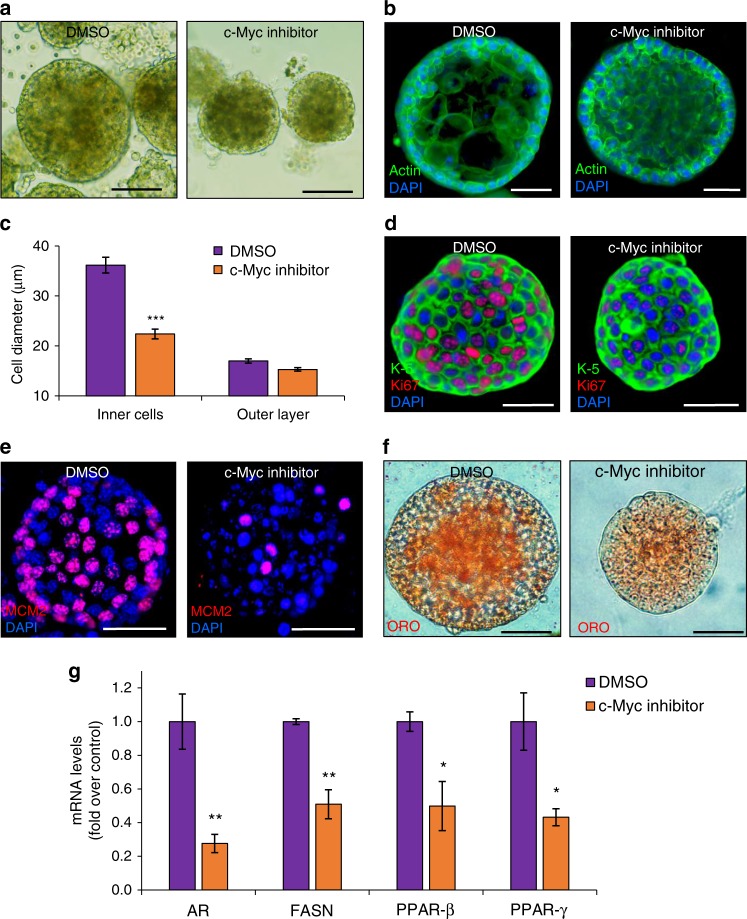
c-Myc regulates proliferation and differentiation in SG organoids. **a** *Blimp1*-YFP^+^-derived sebaceous gland organoids cultured for 6 days and treated for 4 days with c-Myc inhibitor 10058-F4 or DMSO as control. Cross-section of control and treated organoids stained for actin. **b** Treated organoids display decreased organoid size in comparison to control organoids. **c** Cell size analysis of treated and control organoids measuring cell diameter of inner cells and cells at the outer layer [*n* = 80 individual organoids in three independent wells]. **d**, **e** Confocal analysis of treated and control organoids stained with (**d**) Keratin-5 (K-5) and Ki67 and (**e**) MCM2, showing dramatic decrease in proliferating cells upon c-Myc inhibition. **f** Control and treated organoids stained for Oil Red O (ORO), showing diminished differentiation upon c-Myc inhibition. **g** Quantitative real time (RT)–PCR analysis of treated organoids. mRNA levels of known sebocyte differentiation markers including androgen receptor (AR), fatty acid synthase (FASN), and peroxisome proliferator-activated receptors beta and gamma (PPAR-β,ɣ) are significantly decreased upon c-Myc inhibition. Changes in cycle threshold values were normalized to Rplp0. Data are represented as mean relative to control organoids [*n* = 3 independent wells analyzed in triplicates]. Error bars show ± s.e.m. Significance was determined using two-tailed unpaired Student’s *t-*test, where *p < 0.05, **p < 0.005, ***p < 0.001. All images are representative of organoids grown from a mix of at least three mice and cultured in three independent biological repeats. All experiments were repeated at least three times with similar results. Scale bars: 50 μm (**b**, **d**–**f**), 100 μm (**a**)

To monitor the effect of inhibiting c-Myc activity we administered the specific c-Myc inhibitor, 10058-F4, which prevents the c-Myc-Max interaction and inhibits transcription of c-Myc target gene expression^32^. Six-day-old SG organoids were supplemented with the c-Myc inhibitor for 4 days, resulting in a ~40% decrease in organoid size (Fig. 4a, b and Supplementary Fig. 6a). Since the effect on organoid size could result from either altered cell size or number, we examined both these possibilities. Quantifying the diameter of individual cells in the outer proliferating layer of the organoid suggested a modest size decrease (Fig. 4c). However, inhibition of c-Myc had a strong effect on the diameter of cells in the inner compartment, resulting in a ~40% decline in cell diameter size (Fig. 4b, c). In complement, we found that the number of cells in the outer ring of the c-Myc-inhibited organoids had decreased, encompassing cells of smaller size in the inner zone (Fig. 4b, c and Supplementary Fig. 6b, c).

We next examined whether the effect of c-Myc inhibition originates from an effect on cell proliferation and/or differentiation. IF staining against Ki67 and MCM2 indicated a significant decline in Ki67^+^ (69%) and MCM2^+^ (72%) proliferative cells (Fig. 4d, e and Supplementary Fig. 6d). This suggests that proliferation and expansion of the SG organoids is dependent upon c-Myc activity. Our data indicated that, upon c-Myc inhibition, cells in the inner compartment were significantly smaller than their control counterparts, suggesting a possible effect on cell differentiation. Accordingly, c-Myc has been reported as a key regulator of differentiation in the SG in vivo^31, 33^. Initially, we performed lipid staining with the ORO dye, which demonstrated a significant decrease in lipid levels (Fig. 4f and Supplementary Fig. 6e). Using real time (RT)-PCR analysis, we examined mRNA levels of transcripts expressed in SG differentiating or differentiated cells, which play a key role in lipid production^22^. Inhibition of c-Myc decreased the levels of all examined factors, including androgen receptor (AR) and fatty acid synthase (FASN) (Fig. 4g). Furthermore, the central transcription factors peroxisome proliferator-activated receptor-ß and peroxisome proliferator-activated receptor-γ (PPAR-ß and PPAR-γ), which govern numerous genes involved in lipid synthesis and regulation of sebocyte differentiation and maturation^22^, were also significantly attenuated (Fig. 4g). These data show that, like normal SGs, c-Myc plays an instrumental role in regulating both proliferation and differentiation of the Blimp1^+^ cell-derived organoids.

### SG organoids as a platform for studying initial stages of acne vulgaris

We next sought out to examine whether these organoids could be utilized for the study of SG pathologies. Since acne vulgaris effects millions of lives worldwide, we first attempted to generate the conditions where the SG organoids potentially exhibit key aspects of this pathology. Acne vulgaris is characterized by excessive keratin deposition and an increase in SG size, which is dependent upon sebocyte hyperproliferation and sebum production. This serves as an ideal lipid-rich environment for *C. acnes* to flourish which in turn triggers inflammation via the induction of pro-inflammatory cytokines^2^.

**Fig. 5:**
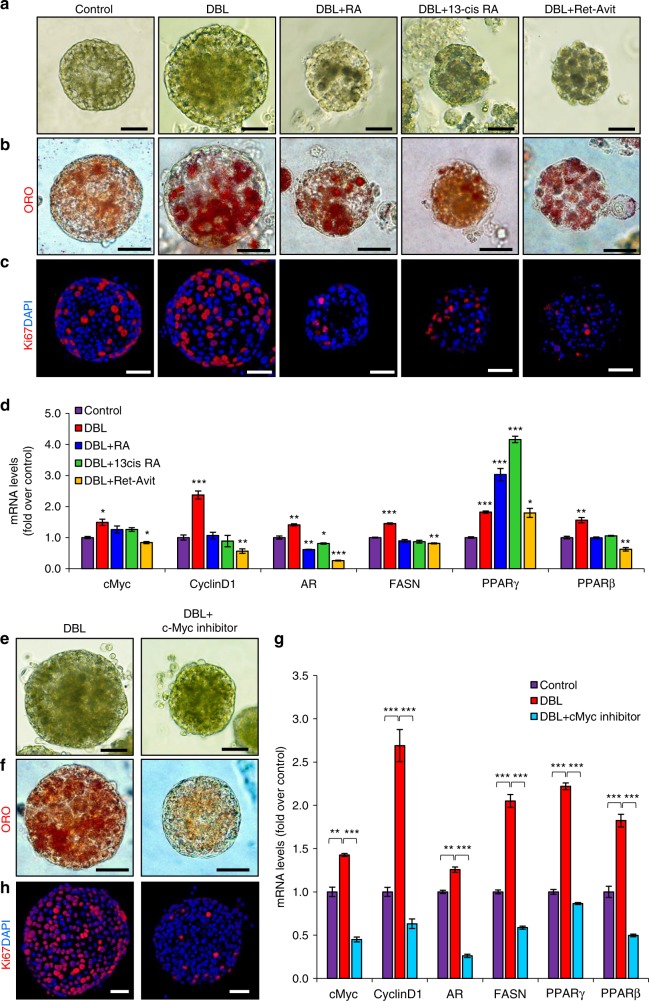
Sebaceous gland organoids can model the initial stages of acne vulgaris. **a**–**d** *Blimp1*-YFP^+^-derived sebaceous gland organoids cultured for 5 days and treated for 3 days with a combination of DHT, BRL, LIN (DBL), or pure ethanol as control, followed by treatments for 3 days with retinoic acid (RA), 13-cis-retinoic acid (13cis RA) or Ret-Avit (Tretinoin). **a** DBL-treated organoids display increased organoid size in compared to control organoids, which is reduced upon treatments with retinoic acid derivatives. **b** Organoids stained for Oil Red O (ORO) show increased lipid production upon DBL treatment and retinoic acid derivatives. **c** Confocal analysis of treated and control organoids stained with Ki67, showing increased proliferation upon androgen induction and decreased proliferation in retinoic acid derivatives-treated organoids. **d** Quantitative real time (RT)-PCR analysis of treated organoids showing changes in mRNA levels of cMyc, Cyclin D1, androgen receptor (AR), fatty acid synthase (FASN), and peroxisome proliferator-activated receptors beta and gamma (PPAR-β,ɣ). **e**–**h** *Blimp1*-YFP^+^-derived sebaceous gland organoids cultured for 5 days and treated for 3 days with DBL combination as before, following treatments for 3 days with c-Myc inhibitor 10058-F4. **e** c-Myc inhibitor-treated organoids display decreased organoid size in compared to DBL-treated organoids. **f** Organoids stained for ORO, showing decreased lipid production upon c-Myc inhibition. **g** Confocal analysis of treated and control organoids stained with Ki67, showing decreased proliferation in c-Myc-inhibited organoids. **h** Quantitative RT-PCR analysis of treated organoids showing changes in mRNA levels of c-Myc, Cyclin D1, androgen receptor (AR), fatty acid synthase (FASN), and peroxisome proliferator-activated receptors beta and gamma (PPAR-β,ɣ). In all RT-PCR experiments, changes in cycle threshold values were normalized to Rplp0. Data are represented as mean relative to control organoids [*n* = 3 independent wells analyzed in triplicates]. Error bars show ± s.e.m. Significance was determined using two-tailed unpaired Student’s *t-*test where *p < 0.05, **p < 0.005, ***p < 0.001. All experiments were repeated at least twice in triplicates with similar results and images are representative of organoids grown from a mix of at least three mice and cultured in three independent biological repeats. Scale bars: 50 μm (**a**–**c**, **e**, **f**, **h**)

Androgen stimulation has been found to play a critical role in regulating sebocyte proliferation and driving the emergence of acne^2^, while PPARs have been shown to alter sebaceous lipid production and modulate acne formation^34, 35^. Therefore, we examined whether we could generate an organoid platform that exhibits key aspects of acne formation, without the presence of *C. acnes* and an inflammatory response, simply by androgen and PPAR stimuli. As a first step, we administered the potent dihydrotestosterone (DHT) androgen, the PPAR-γ BRL-49653 (BRL) activator and linoleic acid (LIN) known to activate PPAR-ß^36^. Administration of BRL, LIN, or DHT for 7 days significantly increased the size of individual SG organoids. While dual combinations did not have an additive effect on organoid size, the combined administration of DHT, BRL, and LIN (denoted DBL) resulted in significantly larger organoids (Fig. 5a, Supplementary Fig. 7a). In accordance, treatment with DBL led to the most considerable increase in mRNA levels of AR, FASN, PPAR-ß, and PPAR-γ, suggestive of increased lipid synthesis (Supplementary Fig. 7b).

In order to assess whether sebum production was indeed affected in response to DBL treatment we performed ORO staining and observed a significant increase in the number of ORO^+^ cells (Fig. 5b and Supplementary Fig. 7c). Of note, as in control organoids and normal SGs, all ORO^+^ cells were located in the inner differentiated part of the DBL-treated organoids (Fig. 5b). To examine whether proliferation was also affected we performed staining against Ki67 and detected a significant increase in the numbers of Ki67^+^ cells upon DBL treatment (Fig. 5c). These results were verified by RT-PCR analysis, indicating the most significant increase in levels of c-Myc and Cyclin D1 transcripts upon DBL treatment (Supplementary Fig. 7d). Taken together our findings indicate that administration of specific androgens and PPAR-stimulators can result in an organoid model that recapitulates initial key aspects of acne development including altered sebocyte proliferation, increase in sebum production, and augmented organoid size.

Since we were able to generate a system that recapitulates key aspects of acne vulgaris, we next examined the extent that currently prescribed medications could affect its development. Retinoids are derivatives of vitamin A, which serve as the first-line treatment for acne^2^. Specifically, all-trans-retinoic acid (ATRA; Tretinoin), formulated ATRA (Ret-Avit), and 13-cis RA (Isotrtinoin) are used worldwide^2^. Administration of all three retinoids to DBL-treated organoids resulted in a significant decrease in organoid size with formulated ATRA yielding the most robust results (Fig. 5b and Supplementary Fig. 7e). Furthermore, retinoid administration instructed cell differentiation leading to a heightened number of ORO^+^ cells (Fig. 5b). Interestingly, in these conditions we could also detect ORO^+^ cells along the SGPZ, suggestive of an altered differentiation program.

The differentiation program in the SG is a multi-step process^33^. Cells prior to the onset or during early stage of differentiation express AR, which is known to reside upstream and lead to the expression of FASN^33^. In response to the different retinol treatments, we could detect a significant decrease in the mRNA levels of AR and FASN, indicating that early differentiating cells were not abundant in these organoids (Fig. 5d). Additionally, the mRNA level of PPAR-ß was also significantly decreased (Fig. 5d). Interestingly, the level of PPAR-γ, which is known to be expressed during middle/late differentiation, was significantly increased (Fig. 5d). This result, in combination with the high numbers and location of ORO^+^ cells, suggests that retinol treatments can advance and alter the differentiation process. In addition to increased differentiation, we found that all retinol treatments led to a drastic decrease in proliferation levels as indicated by Ki67 staining (Fig. 5c and Supplementary Fig. 7f) and RT-PCR analysis examining the mRNA levels of Cyclin D1 and c-Myc.

Retinoids, such as 13-cis RA, are known to have severe side-effects and concerns have been raised regarding the teratogenicity of these compounds^22^. This highlights the need for alternative treatments. Interestingly, a recent genome wide association study of severe teenage acne has suggested c-Myc as a potential regulator^37^. Therefore, we hypothesized that inhibition of c-Myc could serve as a beneficial treatment of acne vulgaris. For this aim, we administered the 10058-F4 c-Myc inhibitor to DBL-treated organoids and monitored size, proliferation, and differentiation. Our results clearly show that c-Myc inhibition results in a significant decrease in organoid size similar to that of retinol treatment (Fig. 5e and Supplementary Fig. 8a). Interestingly, c-Myc inhibition led to a substantial decrease in ORO^+^ cells (Fig. 5f and Supplementary Fig. 8b). Moreover, we found a decrease in the size of individual inner cells (Supplementary Fig. 8c) and significantly lower expression levels of lipid synthesis genes, indicative of diminished differentiation (Fig. 5g). Finally, we monitored cellular proliferation and found a more than 80% decrease in the number of Ki67^+^ cells, correlating to a substantial decrease in the levels of Cyclin D1 and c-Myc transcripts (Fig. 5g and Supplementary Fig. 8d).

Taken together, these results indicate a potential role of c-Myc in the pathogenesis of acne vulgaris and suggest that it may be utilized as a therapeutic target.

## Discussion

In this work, we have generated an organoid model that recapitulates critical aspects of SG biology, which could serve as a tool to study the SG in homeostasis and different pathologies. To achieve this goal, we first set out to establish which cells have the capacity to give rise to a SG mini-organ in vitro. In the past, elegant studies have reported conflicting findings as to which cells within the mouse epidermis express the Blimp1 marker. Initially, it was reported that Blimp1^+^ cells reside exclusively at the base of the SG and serve as a pool of progenitors^3^. Subsequently, it was described that terminally differentiated sebocytes^8^, as well as granular layer IFE keratinocytes^9, 10^ are marked by the Blimp1 protein. Here, by performing advanced 3D confocal analyses and coupling flow cytometry with high-resolution microscopy, we have been able to reconcile these observations and demonstrate that Blimp1 marks cells which reside in each of these locations. Notably, in contrast to terminally differentiated sebocytes and IFE keratinocytes which are post-mitotic, we have been able to expand Blimp1^+^ cells in 2D for extended periods of time, which serves as an indication for their progenitor and stemness features.

We established the in vitro conditions required for the generation of SG organoids by embedding Blimp1^+^ cells in a 3D environment and supplementing the cultures with key factors, known to regulate different aspects of SG biology. These conditions can be easily applied and enable the establishment of SG organoids. Our detailed analyses indicated that Blimp1^+^ cell-derived organoids mimic both the expression profile and homeostatic kinetics of SGs in vivo. Additionally, these organoids encompass lipid-producing cells in the inner mass and by utilizing HPLC coupled with LC/MS we demonstrate that SG organoids display a lipid signature resembling that of SGs in vivo.

A fundamental factor that governs SG expansion in vivo is c-Myc^3, 29–31^^,^. By utilizing SG organoids, we found that c-Myc is only expressed in proliferating SGPZ cells and not in the inner differentiated compartment. Inhibition of c-Myc activity resulted in a significant decrease in cell proliferation and organoid size. Furthermore, c-Myc inhibition had a strong effect on sebocyte differentiation as well as on the size of differentiated inner-organoid sebocytes. These results are in accordance with previously reported findings where overexpression of c-Myc led to enhanced SG expansion, while conditional deletion of *c-Myc* resulted in decreased SG size, cell proliferation, and sebocyte differentiation^3, 29, 38, 39^. Notably, Blimp1 has been shown to govern the size of SGs by repressing *c-Myc* gene expression^3^. Thus, it will be interesting to examine which additional factors can regulate the activation and expression of c-Myc. As SG organoids capture the complex function of c-Myc, we hypothesize that this platform can be utilized for investigating various molecular circuits governing SG homeostasis and development.

Acne vulgaris is a chronic disease of the pilosebaceous unit resulting from androgen-induced increased sebum production^40^. Some of the key features of acne development include disturbed SG activity resulting in excessive sebum, altered sebocyte proliferation and differentiation, dysregulation of the hormonal environment, hyperkeratinization, colonization of *C. acnes* and inflammation^2, 40^.

Utilizing our SG platform we were able to generate features of acne. We administered the organoids with PPAR activators and the potent androgen, DHT, which are known to lead to hyperseborrohoea and increased sebcote proliferation^2^. This resulted in organoids that grew substantially in size and were characterized by excessive sebum production, increased sebocyte proliferation, and altered differentiation.

Of note, key aspects of acne vulgaris include *C. acnes* colonization and the induction of an inflammatory response which are not present in our model. In this regard, since it has been shown that *C. acnes* upregulates the expression of pro-inflammatory cytokines in cultured sebocytes^2^, it may be interesting to examine such an effect in the organoid platform. As additional bacterial strains are suspected to play a role in the development of acne^41^, our system enables examination of their function and contribution to this process.

It has been proposed that, as acne vulgaris is solely human disease, it cannot be recapitulated by animal models. As we have utilized mouse cells for establishing the SG organoid platform and it lacks important stages in acne development, it is key to note that it does not fully recapitulate a human model. Nevertheless, we show that administration of currently available acne treatments affected SG organoid size, proliferation, and differentiation, akin to acne in vivo. Furthermore, this platform indicated that c-Myc plays an important role in the pathology of acne as recently suggested by a genome wide screen^37^. Our findings suggest that c-Myc can potentially be used as a therapeutic target for the treatment of acne thus indicating that our SG model can potentially serve for the discovery of previously undescribed acne therapeutics.

In summary, our findings bring forward a platform that may be utilized for drug screening purposes, as well as for studying SG homeostasis, functions, and pathologies.

## Methods

### Mice

All animal studies gained approval by the Committee on the Ethics of Animal Experiments of the Technion-Israel Institute of Technology. *B6.Cg-Tg(Prdm1-EYFP)1Mnz/J* mice were purchased from Jackson. All mice were bred and housed in the Technion animal facility under specific pathogen-free conditions. Mice were provided access to food and water ad libitum. Mice were sacrificed using CO_2_ intoxication, in accordance with our institutional guidelines at 8 weeks of age. Mice of both sexes were utilized for all analyses.

### Flow cytometry

Dorsal skin from 8-week-old mouse was shaved and harvested. Underlying adipose tissue was removed before incubting in trypsin overnight at 4 °C or 2 h at 37 °C^42^. Epidermis and hairs were collected and filtered before sorting^43^. Isolation of α6^+^ScaI^−^Blimp-YFP^+^ cells was performed using a BD FACSAria IIIu sorter, sorting for endogenous *Blimp1*-YFP^+^ and utilizing antibodies against Integrin α6 (eBioscience, #12-0495-82) and ScaI (BD Pharmigen, #558162). Amnis ImageStreamX Mark II Flow cytometer was used to visualize *Blimp*-YFP^+^-sorted cells.

### Cell culture

α6^+^ScaI^−^Blimp-YFP^+^ cells were cultured in keratinocyte stem cell medium^44^. Briefly, medium is prepared using Dulbecco’s modified Eagle medium (DMEM)/F12 3:1 (Biological Industries) containing l-glutamine (Biological Industries; 1:100), penicillin/streptomycin (Biological Industries; 1:100), 10% chelated fetal bovine serum, 5 µg/ml insulin (Sigma I-5500), 5 µg/ml transferrin (Sigma T-2252), 2 × 10^−12^ M T3 (3,3′-triiodo-l-thyronine; Sigma T-2752), 400 ng/ml hydrocortisone (Sigma H0888), cholera toxin 10^−10^ M and 50 mM CaCl_2_. Cells were grown on sustaining J2 feeder cells for 4–6 passages until stable colonies were formed, followed by culturing without feeders. For organoid generation a total of ~5000 cells were mixed with 50 μl of Matrigel (BD Bioscience) and plated in 24-well plates. 500 μl of keratinocyte stem cell medium media containing growth factors: 20 ng ml^−1^ hFGF (Peprotech), 40 ng ml^−1^ mEGF (Peprotech), 500 ng ml^−1^ hR-spondin 1, 100 ng ml^−1^ mNoggin (Peprotech), and B-27 Supplement (50×; Invitrogen) was added after polymerization of Matrigel.

Treatment with c-Myc inhibitor 10058-F4 (100 µM, Tocris Bioscience) or DMSO (as a control) was performed for 4 days and renewed upon media change.

5-Bromo-2′-deoxyuridine (BrdU; 1:1000 dilution, GE Healthcare) pulse-chase experiments were performed by treating 6-day-old organoids for 8 h. For analyses, organoids were fixed in 4% PFA for 1 h for IF or harvested for RNA extraction.

In order to establish conditions mimicking acne vulgaris, SG organoids cultured for 5 days and treated for 3 days with DHT 10^−5^ M, BRL 10−6 M, LIN 10^−4^ M (Sigma Aldrich), or pure ethanol as control, following treatments for 3 days with retinoic acid 100 µM (Sigma Aldrich), 13-cis-retinoic acid 100 µM (Sigma Aldrich), or Ret-Avit (Tretinoin 0.05% w/w, final 10 µM) (CTS Chemical industries Ltd.)

### Live imaging

*Blimp1-*YFP^+^ cells were infected with retroviruses LV-GFP (Addgene, Plasmid #25999), which fluorescently labels the nuclear histones. From these, infected organoids were generated for 7 days, followed by visualization using light sheet fluorescence microscopy (LSFM) for a duration of 24 h. Analyses of the data including cells division tracking was performed using Imaris Image Analysis Software.

### Histology and IF

For whole mount preparation, tail skin samples were treated with 20 mM EDTA for 4 h at 37 °C to separate skin epithelium from dermis and then fixed in 4% formaldehyde for 2 h at room temperature. Dorsal skin samples were treated with 20 mM EDTA for 6 h at 37 °C to separate skin epithelium from dermis and fixed in 4% formaldehyde for 1 h at room temperature.

Following fixation, samples were blocked for 2 h in blocking buffer consisting of 10% goat serum, 2% BSA, and 1% Triton-X. Primary antibodies were diluted in blocking buffer and samples were incubated overnight at 4 °C. Samples were washed at least three times with PBS. Secondary antibodies were incubated for 1 h at room temperature followed by four washes with PBS.

The following primary antibodies were used: Ki67 (rabbit, 1:100, Abcam; rat, 1:100, eBioscience), MCM2 (rabbit, 1:500, Abcam), BrdU (mouse, 1:200, Santa Cruz), c-Myc (mouse, 1:100, Santa Cruz), Phalloidin (1:250, Life Technologies), K-15 (mouse, 1:100, Thermo; chicken, 1:1000, Abcam), and K-5 (rabbit, 1:500, Abcam).

Secondary antibodies conjugated to Alexa Fluor-488, Alexa Fluor-546, and Alexa Fluor-633 were used in order to visualize the primary antibodies. For lipid visualization, Oil-red-O (ORO) staining was performed by incubating samples in 0.18% ORO in 60%isopropanol for 20 min, followed by three washes with PBS. Imaging analyses were performed on a Zeiss LSM 880 confocal microscope.

### RNA extraction and real-time PCR

RNA isolation was performed using TRIzol reagent according to manufactures protocol (Sigma). Up to 2 μg of RNA were used for cDNA production (Applied Biosystems). Quantitative real time (qRT)-PCR was performed using the PerfeCTa SYBR Green FastMix (Quanta), with primers specific for the following genes: RpLp0: GCGACCTGGAAGTCCAACTA and ATCTGCTTGGAGCCCACAT3, AR: AGAATCCCACATCCTGCTCAA and AAGTCCACGCTCACCATATGG, PPAR-gamma: GATGGAAGACCACTCGCATT and AACCATTGGGTCAGCTCTTG, PPAR-beta: TTCCTTCCAGCAGCTGTG and TCGCACGCGTGGACC, FASN: CCCTTGATGAAGAGGGATCA and ACTCCACAGGTGGGAACAAG, cMyc: TAGTGCTGCATGAGGAGACA and CATCAATTTCTTCCTCATCTTC, Cyclin-D1: ATTGTGCCATCCATGCG and TAGATGCACAACTTCTCGGC). Levels of specific amplicons were analyzed in three biological repeats and quantified relative to a standard curve comprising serial cDNA dilutions. The mRNA values were normalized to housekeeping gene (Rplp0). Cycling parameters: initial denaturation for 3 min at 95 °C, following 40 cycles of: denaturation for 10 s at 95 °C; annealing, extension, and read fluorescence for 30 s at 60 °C; addition of melt curve step: 10 s at 95 °C, with increments of 0.5 °C every 5 s between 65 and 95 °C.

### Lipidomics sample preparation

Solvents for sample preparation, as well as for chromatography (LC/MS grade acetonitrile, isopropanol, methanol, water, HPLC grade chloroform) were purchased from Bio-Lab, Inc. Solvent composition is provided as volume-to-volume ratio. Lipids were extracted with the 2:1 chloroform:methanol solution according to the Folch method^28^. Phase separation in the extract was achieved by the addition of equivalent water volume and centrifugation at 2000×*g* for 5 min. The upper and middle phases were removed by aspiration and the lower phase was dried in the nitrogen flow. Lipid extract was re-dissolved in 1:1 isopropanol:methanol and stored for analysis.

### LC/MS data acquisition

For the reversed-phase chromatographic separation, Phenomenex Kinetex C-18-XB 150 mm column (3 mm i.d., 2 µm beads) was used. The column compartment of HPLC was maintained at 65 °C, sample vials at 30 °C. Mobile phase was composed of two solvent mixes as follows: A—60:40 acetonitrile:water mix having 10 mM ammonium formate (HPLC grade, Sigma), B— 90:8:2 acetonitrile:isopropanol:water with 10 mM ammonium formate. For the solvent B, ammonium formate was dissolved in corresponding water volume before mixing with the organic solvents. The buffer loop in the autosampler was filled with 1:1 isopropanol:methanol. Mobile phase flow was 0.5 mL/min. Mobile phase composition was changing according to the following program with linear gradients: 0 min—15% B; 2 min—30% B; 2.5 min—48% B; 11 min—82% B; 11.5 min—99% B, maintained until 14 min, and then switched back to 15% B and equilibrated for 2.5 min. Chromatographic eluent was analyzed by ESI–MS Thermo Orbitrap Q Exactive mass spectrometer. Ion source supplied 50 a.u. of sheath gas flow and 20 a.u. of auxiliary gas flow. Auxiliary gas and the ion transfer capillary were heated to 350 °C. S-Lens RF level was 80 a.u. Analysis was performed in polarity switching mode with the negative mode voltage −3.5 kV, and the positive mode voltage +3.3 kV. Automatic gain control was tuned to 10^6^, scanning range was from 120 to 1800 *m*/*z* with mass resolution 70,000 in both polarity modes. Tandem mass spectra for the corresponding samples were acquired in the separate technical repeats with the following parameters of data-dependent acquisition: isolation width 1.4 Da, maximum ion accumulation time prior to fragmentation 600 ms, normalized collision energy 30 a.u., minimum AGC target 10^4^, fixed first mass 50 Da, three fragmentation spectra per one MS1 scan, multiple-charge ions exclusion, resolution 17,500 for MS1 and MS2 scans, no polarity switching.

### Lipidomics

According to the LC/MS method, two primary types of ions were detected in both polarity modes: protonated analytes and NH_4_^+^ adducts in the positive mode; deprotonated analytes and HCOO^−^ adducts in the negative mode. Peak peaking and intensity calling were performed in XCMS^45, 46^. Stable reproducibility of chromatograms allowed no peak alignment. A custom-made R script was used to identify M + 0 peaks. For further analysis, only M + 0 peaks of lipid compounds were considered, with the intensity not less than 5 × 10^5^ in at least one of samples. For lipid mass list, lipids from Lipid Mass Structure Database^47^ were used together with a list of compounds created by a combination of hydrophilic “head” structures with even-numbered fatty acids. Selected groups of peaks and their natural carbon mass-isotopomer distributions were verified manually in Maven software^48, 49^. Intensities in each sample were log-transformed and converted to *z*-scores. Additionally, to compare intensity profiles between compounds, second normalization to *z*-scores was applied to each row.

PCA was performed on normalized dataset. Together, first four principal components represented 81% of variance. Space of these four principal components, each representing more than 10% of total variance, was used for clustering of peak profiles. Clustering was performed with *k*-means algorithm for three centers. Resulted clusters were attributed to specific groups of samples according to the maximal intensity in the averaged profile.

### Statistical analyses

At least three mice were analyzed in in vivo experiments. Organoid were grown from a mix of at least three mice and cultured in three independent biological repeats. All experiments were repeated at least twice. Statistical analyses were performed using two-tailed unpaired Student’s *t* test, where *p < 0.05, **p < 0.005, ***p < 0.001. All quantifications display the mean of the data and error bars represent ± s.e.m. Images were processed and analyzed using the Imaris, ImageJ, FSC Express Flow Cytometry Data Analysis, and ZEN programs.

### Reporting summary

Further information on research design is available in the Nature Research Reporting Summary linked to this article.

## Supplementary information

Supplementary Information

Supplementary Movie 1

Supplementary Movie 2

Reporting Summary

Description of Additional Supplementary Files

Source Data

## Data Availability

The authors declare that all data supporting the findings of this study are available within this article and its supplementary information files or from the corresponding author upon reasonable request. The source data underlying Figs. 4c, 4g, 5d, 5g and Supplementary Figs. 1e, 1j, 2a-b, 6a, 6b, 6d, 7a, 7b+d, 7c+8b, 7e+8a, 7f+8d, 8c are provided as a Source Data file.
