# Supplementary material for: Blimp1+ cells generate functional mouse sebaceous gland organoids in vitro
Source: Nat Commun. 2019 May 28;10:2348. doi: 10.1038/s41467-019-10261-6 (PMC6538623; doi:10.1038/s41467-019-10261-6)
Supplement: Supplementary file 1 — Supplementary Information [file 41467_2019_10261_MOESM1_ESM.pdf]

Supplementary Information

Blimp1$^+$ cells generate functional mouse sebaceous gland organoids *in vitro*

Feldman *et al.*

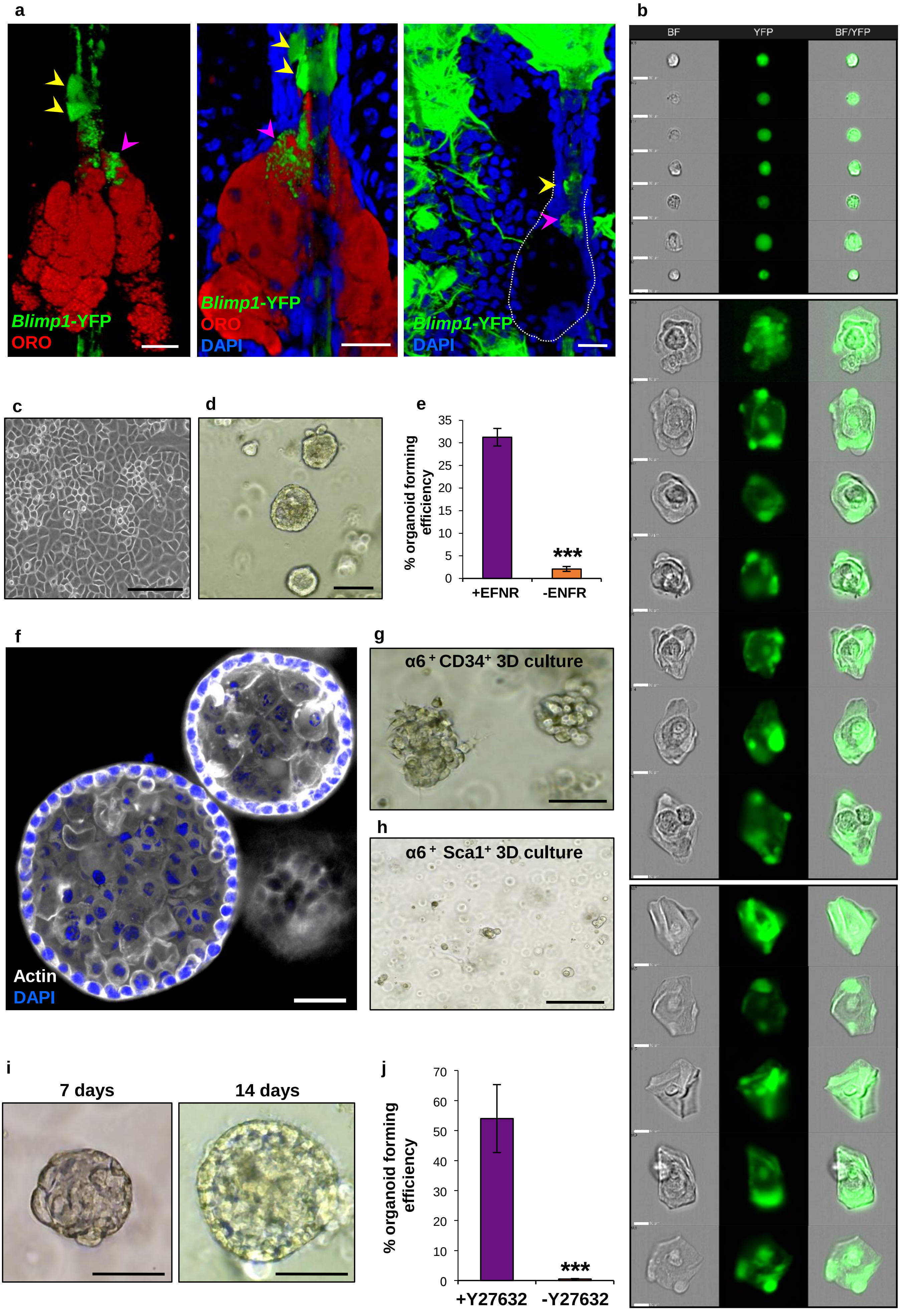
a
Blimp1-YFP
ORO
Blimp1-YFP
ORO
DAPI
Blimp1-YFP
DAPI
b
BF
YFP
BF/YFP
c
d
e
% organoid forming efficiency
35
30
25
20
15
10
5
0
***
+EFNR
-ENFR
f
Actin
DAPI
g
α6 + CD34+ 3D culture
h
α6 + Sca1+ 3D culture
i
7 days
14 days
j
% organoid forming efficiency
70
60
50
40
30
20
10
0
***
+Y27632
-Y27632

Supplementary Figure 1. *Blimp1*-YFP^{+} cells can generate a sebaceous gland-resembling structure in 3D culture. (**a**) Representative images of dorsal skin whole-mount extracted from P56 (telogenic)-old *B6.Cg-Tg(Prdm1-EYFP)1Mnz/J* (denoted *Blimp1-YFP*) reporter mouse stained for Oil Red O (ORO) shows *Blimp1*-YFP^{+} expression at the base of the sebaceous gland (SG; yellow arrows), in mature sebocytes (pink arrow), and differentiated keratinocytes in the interfollicular epidermis [*n* = 3 mice]. (**b**) Representative ImageStream analysis showing brightfield (BF) images of single *Blimp1*-YFP^{+} cells, indicating that these cells display three distinct morphologies: 1) small cells with high circularity, 2) large cells resembling sebocytes and 3) differentiated keratinocytes. (**c**) 2D culture of α6^{+};ScaI^{-};*Blimp1*-EYFP^{+} cells at passage 10 [*n* = 3 pooled mice]. (**d**) 3D culture of α6^{+};ScaI^{-};*Blimp1*-EYFP^{+} cell-derived organoids grown in Matrigel without supplemented EGF, FGF, Noggin and R-spondin (EFNR) medium. (**e**) Organoid forming efficiency of cultured α6^{+};ScaI^{-};*Blimp1*-EYFP^{+} cells, grown in the presence or absence of EFNR media. (**f**) 3D-reconstructed confocal image of a cross-section obtained from a day 10 organoid, stained for actin. (**g, h**) 3D culture of (**g**) α6^{+};CD34^{+} and (**h**) α6^{+};ScaI^{+} cells in Matrigel. (**i**) 3D culture of isolated α6^{+};ScaI^{-};*Blimp1*-EYFP^{+} cells directly seeded in Matrigel, supplemented with EFNR and ROCK inhibitor Y-27632 after 7 and 14 days. (**j**) Organoid forming efficiency from isolated α6^{+};ScaI^{-};*Blimp1*-EYFP^{+} cells directly seeded in Matrigel, supplemented with EFNR and grown in the presence or absence of ROCK inhibitor Y-27632. All organoids images are representative of a mix of at least three mice, cultured in three independent biological repeats. Experiments were repeated at least twice with similar results. Error bars represent ±s.e.m. ***p<0.001 was determined using two-tailed, unpaired Student's *t* test. Scale bars: 10μm (**b**), 20μm (**a**) 50μm (**c, d, f-i**).

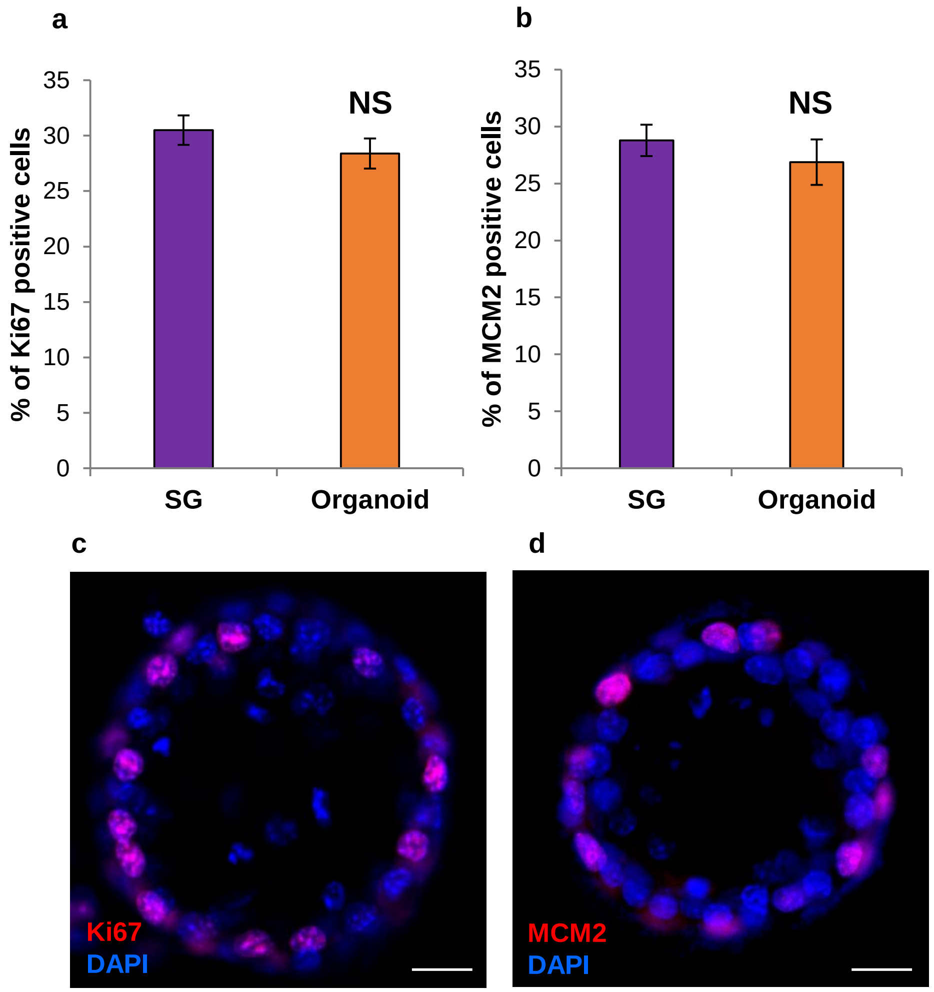

Supplementary Figure 2. Proliferation in sebaceous glands and *Blimp1*-YFP⁺ cell-derived organoids. (**a, b**) Percentages of (**a**) Ki67⁺ and (**b**) MCM2⁺ cells in sebaceous glands (SGs) of P56 (telogenic)-old wild type mice and *Blimp1*-YFP⁺ cell-derived organoids that were cultured for 10 days. Error bars represent ±s.e.m [*n* = 3 mice or 60 individual organoids from three independent wells]. NS indicates no significance as determined by two-tailed, unpaired Student's *t* test. (**b, c**) Representative images of cross-section of 10-day-old organoid stained with (**a**) Ki67 and (**b**) MCM2, showing proliferating cells only on the outer organoid layer. Experiments were repeated at least three times with similar results. Scale bars: 20μm (**c, d**).

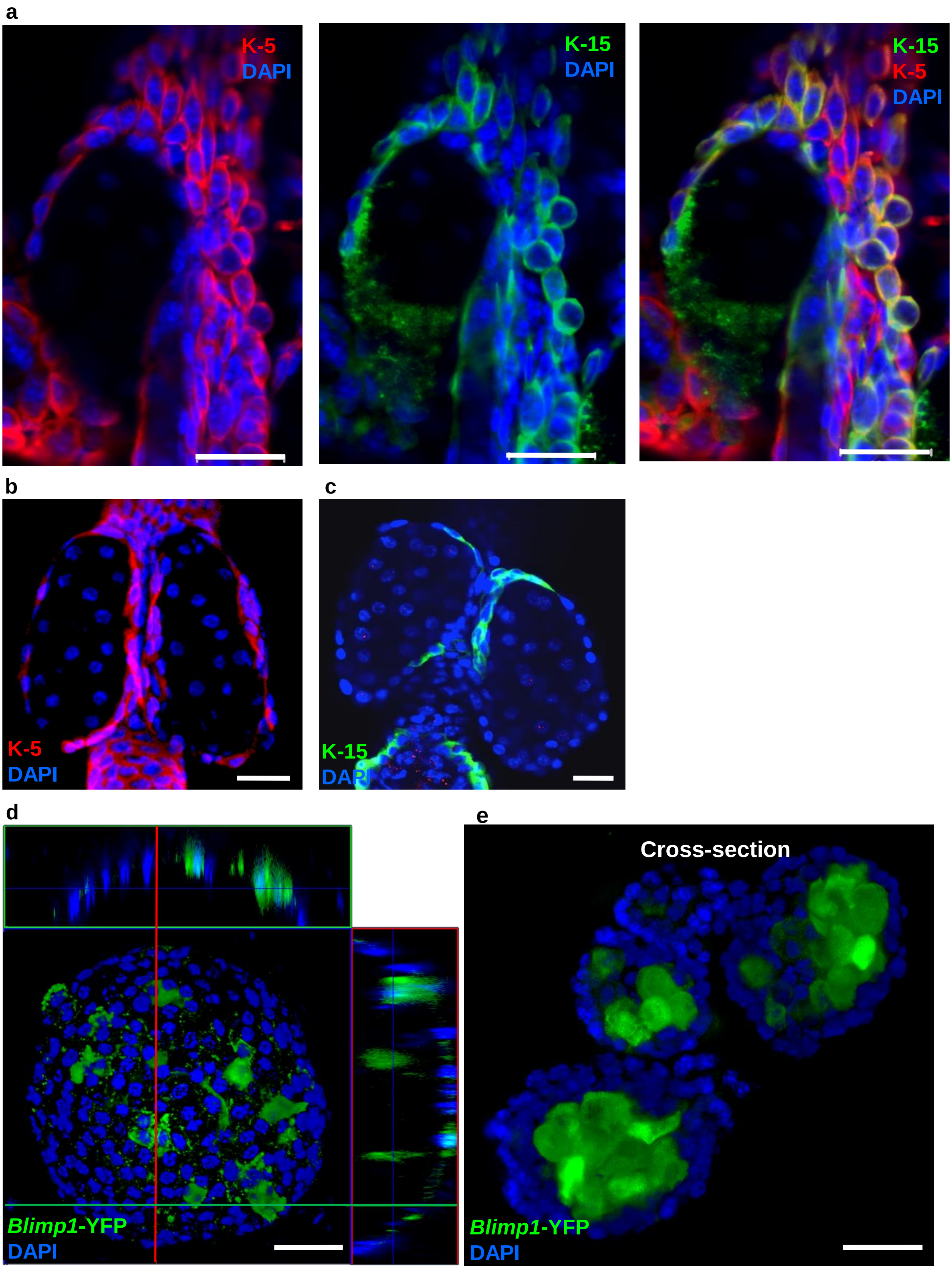

Supplementary Figure 3. Expression of sebaceous gland markers in sebaceous glands and organoids. (**a**) Dorsal skin whole mount extracted from P56 (telogenic)-old wild type (WT) mouse stained for keratin (K)-5 and K-15. (**b, c**) Tail skin wholemount extracted from P56 (telogenic)-old WT mouse stained for (**b**) K-5 and (**c**) K-15. (**d, e**) Confocal images of *Blimp1*-YFP^{+}-cell derived organoids cultured for 10 days, showing (**d**) *Blimp1*-YFP^{+} cells in the outer layer and (**e**) in the inner compartment as shown in an organoid cross-section. All images are representative of three mice or organoids derived from three pooled mice cultured in triplicates. Experiments were repeated at least three times with similar results. Scale bars: 20μm (**a-c**), 50μm (**d, e**).

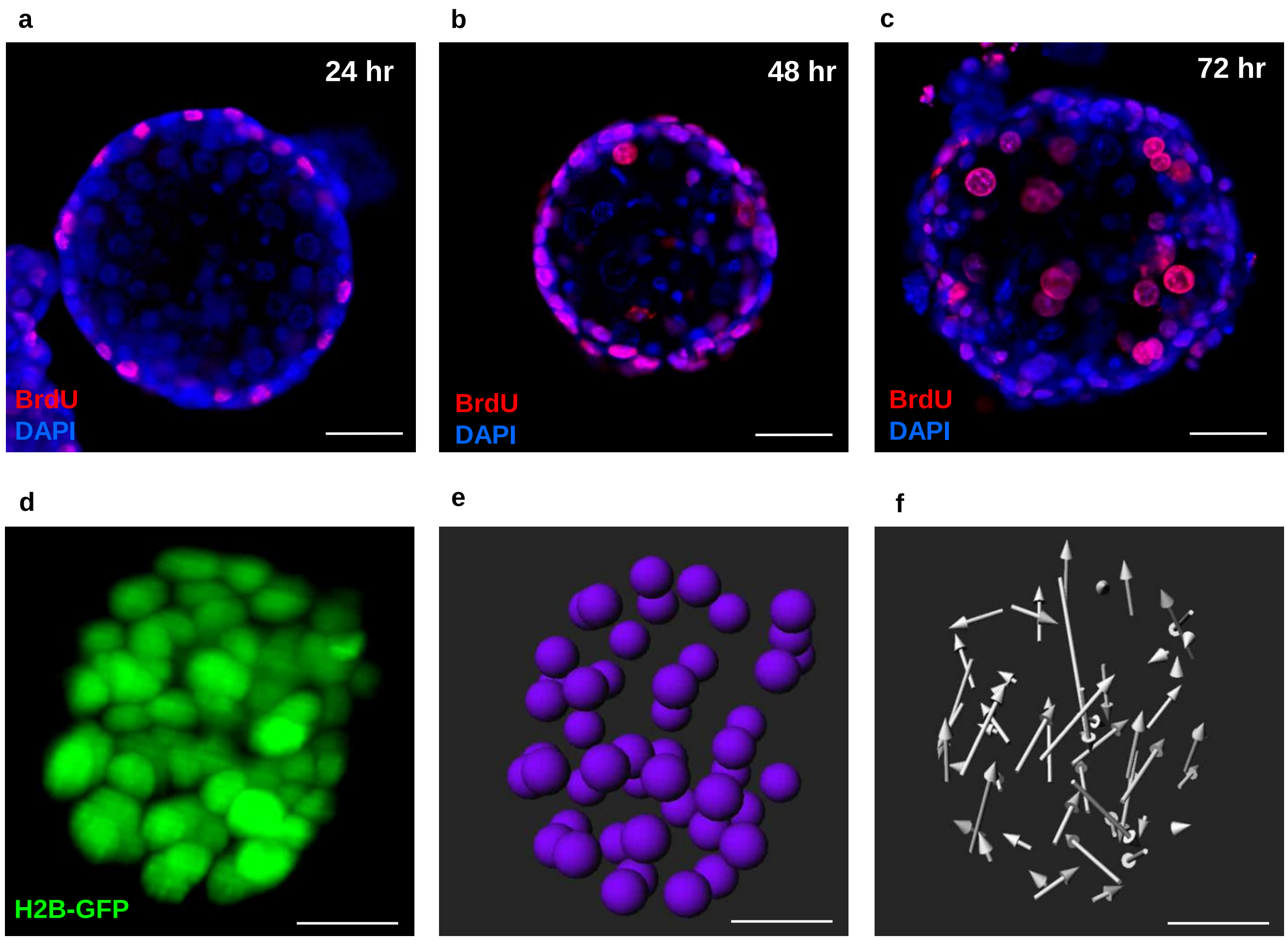

Supplementary Figure 4. Analysis of cell dynamics in sebaceous gland organoids. (**a-c**) 5-Bromo-2′-deoxyuridine (BrdU) pulse-chase analysis in 7-day-old *Blimp1*-YFP^{+} cell-derived organoids, chased over (**a**) 24 hours, (**b**) 48 hours and (**c**) 72 hours. Cross-sections revealing the migration of BrdU-labeled cells from the outer layer to the inner non-proliferating mass. (**d**) Light sheet fluorescence microscopy image of representative *Blimp1*-YFP-H2B-GFP^{+} (nuclear labeled) cell-derived organoid grown for 7 days. (**e**) Analysis of cell movement was performed and each nucleus is marked as a spheroid. (**f**) Spatial movement was tracked and is represented as a vector. All images are representative of organoids derived from three pooled mice cultured in triplicates. Experiments were repeated at least three times in triplicates with similar results. Scale bars: 20μm (**d-f**), 50μm (**a-c**).

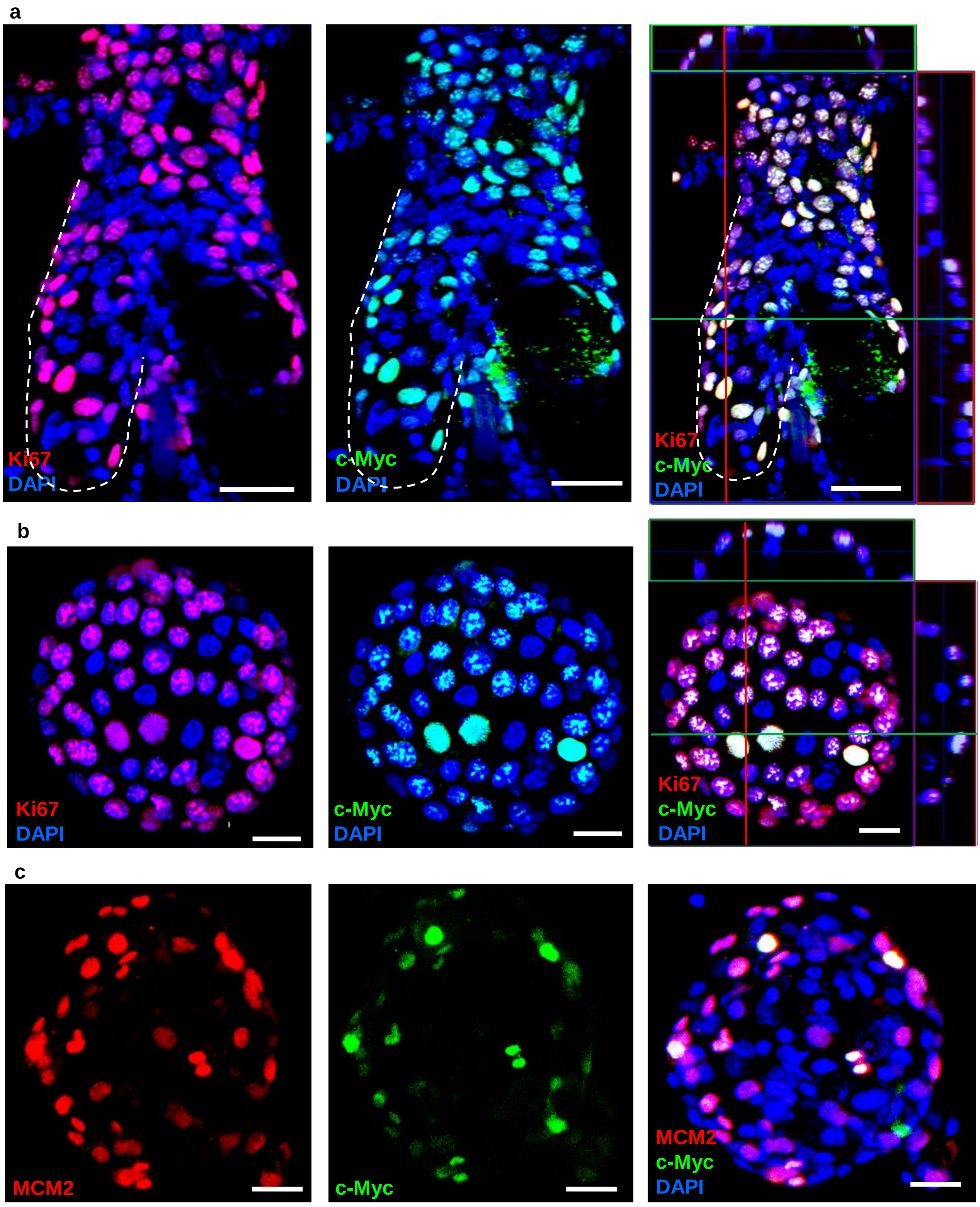

Supplementary Figure 5. c-Myc expression in proliferative cells is recapitulated in organoids. (**a**) Representative images of dorsal skin whole mount extracted from P56 (telogenic)-old wild type (WT) mouse stained for Ki67 and c-Myc [*n* = 3 mice]. (**b, c**) Representative images of *Blimp1*-YFP^{+} cell-derived organoids cultured for 10 days, stained for (**b**) Ki67 and c-Myc and (**c**) MCM2 and c-Myc. Organoids were grown from three pooled mice and cultured in triplicates. Experiments were repeated at least twice with similar results. Scale bars: 20µm (**a-c**).

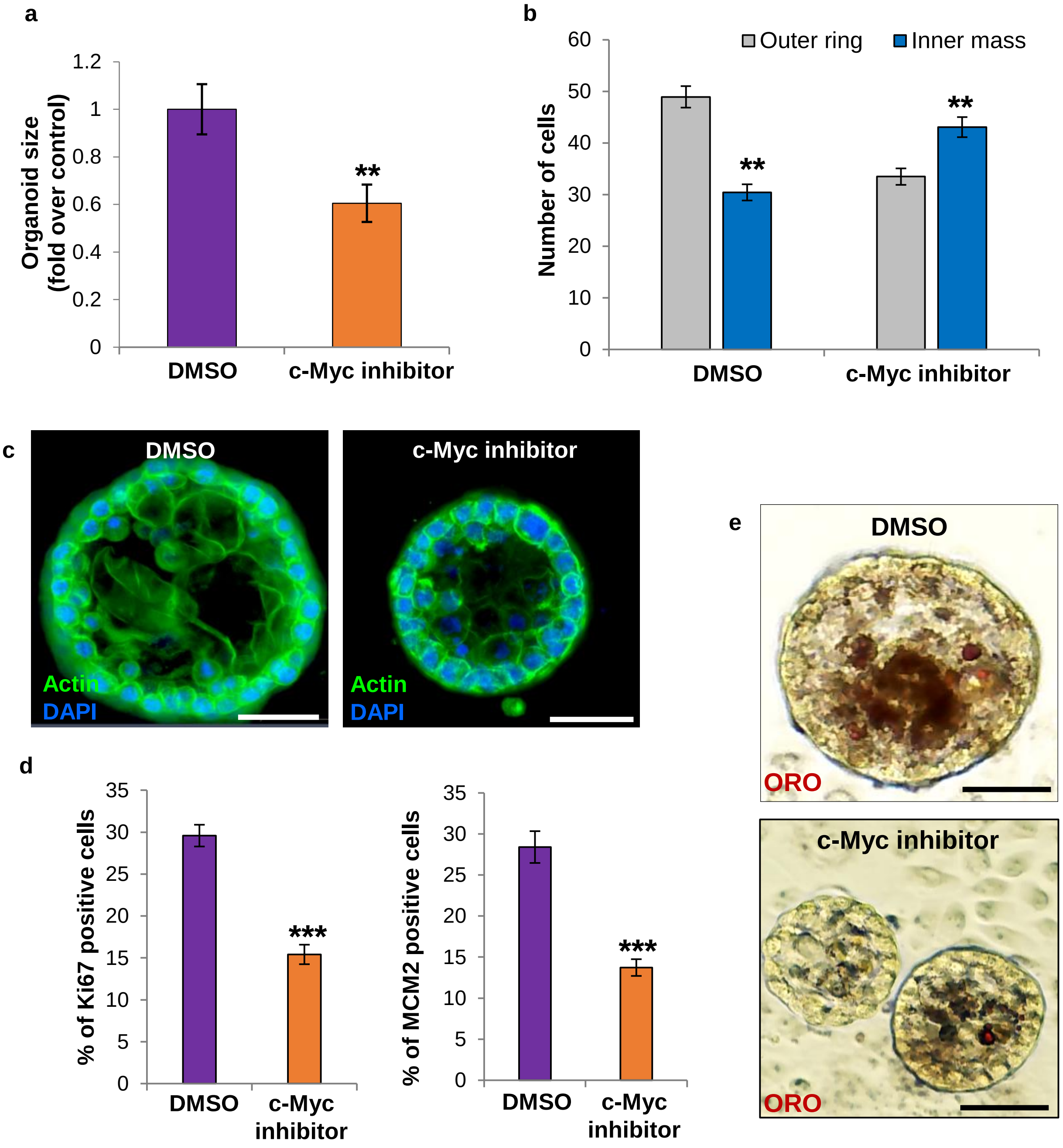

Supplementary Figure 6. c-Myc regulates proliferation and differentiation in the *Blimp1*-YFP⁺ cell-derived organoid. (**a, b**) *Blimp1*-YFP$^+$ cell-derived organoids cultured for six days and treated for four days with c-Myc inhibitor (10058-F4) or DMSO as control. (**a**) Organoid size in treated organoids compared to control organoids (n=150 organoids). (**b**) Cell number in the outer layer and inner mass of control and treated organoids (n=60). (**c**) Cross-section of control and treated organoids stained for actin. (**d**) Percentage of proliferating cells, as marked by Ki67 and MCM2, per control or treated organoid (n=60). (**e**) Control and treated organoids stained for Oil Red O (ORO). Data is represented as the mean and error bars show ±s.e.m relative to control organoids. **p<0.005 and ***p<0.001 were determined using two-tailed, unpaired Student's t test. All images are representative of organoids derived from three pooled mice cultured in triplicates and experiments were repeated at least three times with similar results. Scale bars: 50μm (**c, e**).

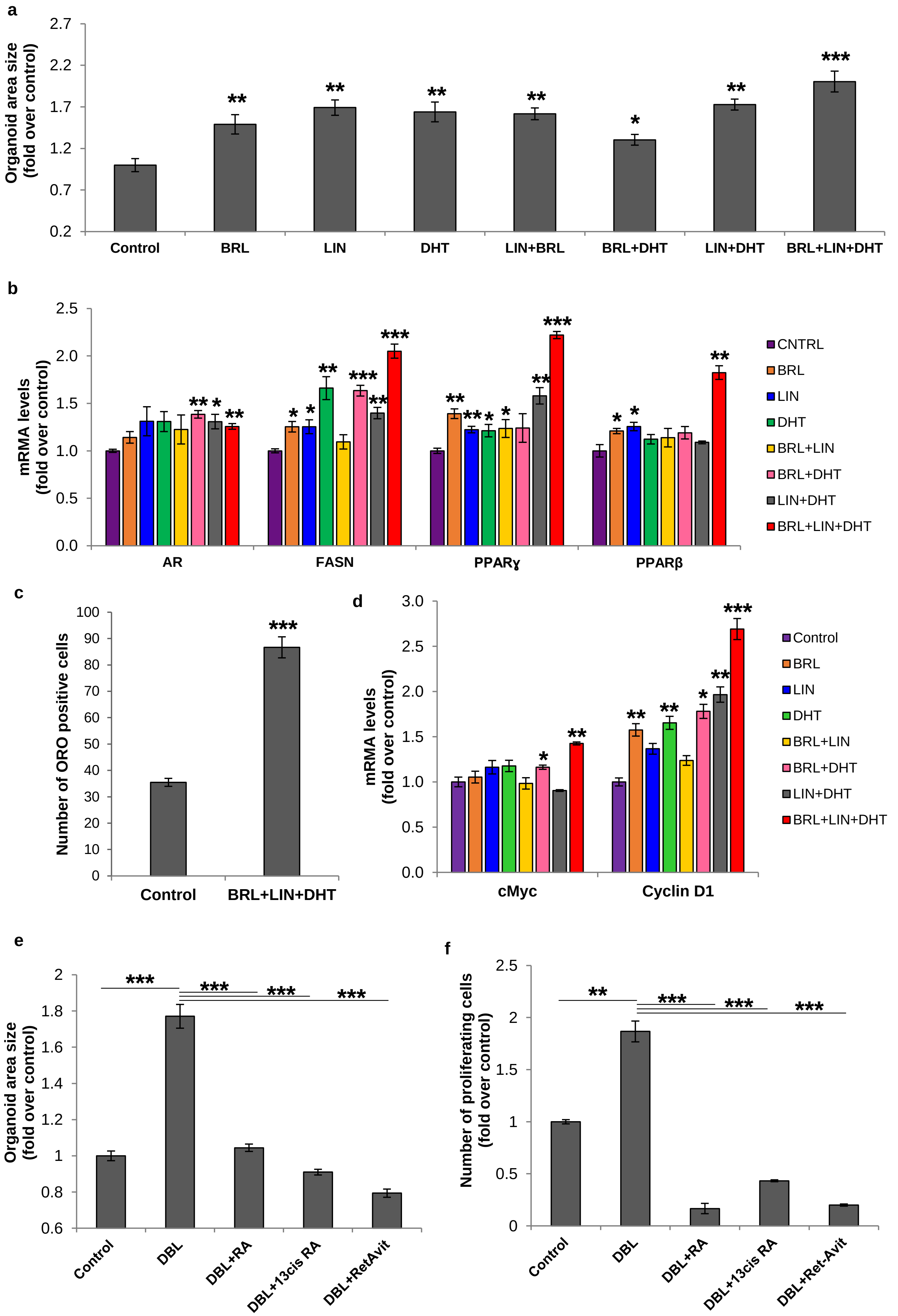
a
Organoid area size (fold over control)
2.7
2.2
1.7
1.2
0.7
0.2
Control
BRL
LIN
DHT
LIN+BRL
BRL+DHT
LIN+DHT
BRL+LIN+DHT
b
mRMA levels (fold over control)
2.5
2.0
1.5
1.0
0.5
0.0
AR
FASN
PPARɣ
PPARβ
CNTRL
BRL
LIN
DHT
BRL+LIN
BRL+DHT
LIN+DHT
BRL+LIN+DHT
c
Number of ORO positive cells
100
90
80
70
60
50
40
30
20
10
0
Control
BRL+LIN+DHT
d
mRMA levels (fold over control)
3.0
2.5
2.0
1.5
1.0
0.5
0.0
cMyc
Cyclin D1
Control
BRL
LIN
DHT
BRL+LIN
BRL+DHT
LIN+DHT
BRL+LIN+DHT
e
Organoid area size (fold over control)
2
1.8
1.6
1.4
1.2
1
0.8
0.6
Control
DBL
DBL+RA
DBL+13cis RA
DBL+RetAvit
f
Number of proliferating cells (fold over control)
2.5
2
1.5
1
0.5
0
Control
DBL
DBL+RA
DBL+13cis RA
DBL+Ret-Avit

Supplementary Figure 7. Sebaceous gland organoids can be used as a model for initial stages of acne vulgaris. (**a-d**) *Blimp1*-YFP^{+} cell-derived organoids cultured for seven days and treated for three days with 100 μM BRL-49653 (BRL), 100 μM Linoleic acid (LIN) or 100 μM Dihydrotestosterone (DHT), the combinations of them pairwise, all of them together or pure ethanol as control. (**a**) Statistical analysis of treated organoids size compared to control organoids [*n* = 150 organoids from 3 independent wells]. (**b, c**) Quantitative real time (RT)–PCR analysis of treated organoids. (**b**) mRNA levels of androgen receptor (AR), Fatty Acid Synthase (FASN), peroxisome proliferator-activated receptors gamma (PPAR-ɣ) and beta (PPAR-β) are significantly increased upon combined treatment (DBL). (**c**) Number of Oil red O (ORO)$^{+}$ cells in combined DBL treatment compared to control organoids [*n* = 120 individual organoids from 3 independent wells]. (**d**) mRNA levels of cMyc and Cyclin-D1 are significantly increased upon combined treatment. (**e, f**) *Blimp1*-YFP^{+}-derived sebaceous gland organoids cultured for five days and treated for three days with a combination of DHT 10^{-5}M, BRL 10^{-6}M, LIN 10^{-4}M (DBL), or pure ethanol as control, followed by treatments for three days with Retinoic acid (RA) 100 μM, 13-cis-Retinoic acid (13cis RA) 100 μM or Ret-Avit (Tretinoin 0.05% final 10 μM). (**e**) DBL-treated organoids display increased organoid size in comparison to control organoids, which is reduced upon treatments with Retinoic acid derivatives. [*n* = 120 individual organoids from 3 independent wells] (**f**) Number of Ki67^{+} proliferating cells indicates increased proliferation in DBL-treated organoids, which is significantly reduced upon treatments with Retinoic acid derivatives [*n* = 60 individual organoids from 3 independent wells]. In RT-PCR experiments, changes in cycle threshold values were normalized to Rplp0 [*n* = 3 independent wells analyzed in triplicates]. Data show the mean relative to controls and error bars represent ±s.e.m. *p<0.05 **p<0.005, ***p<0.001 as determined by two-tailed, unpaired Student's *t* test.

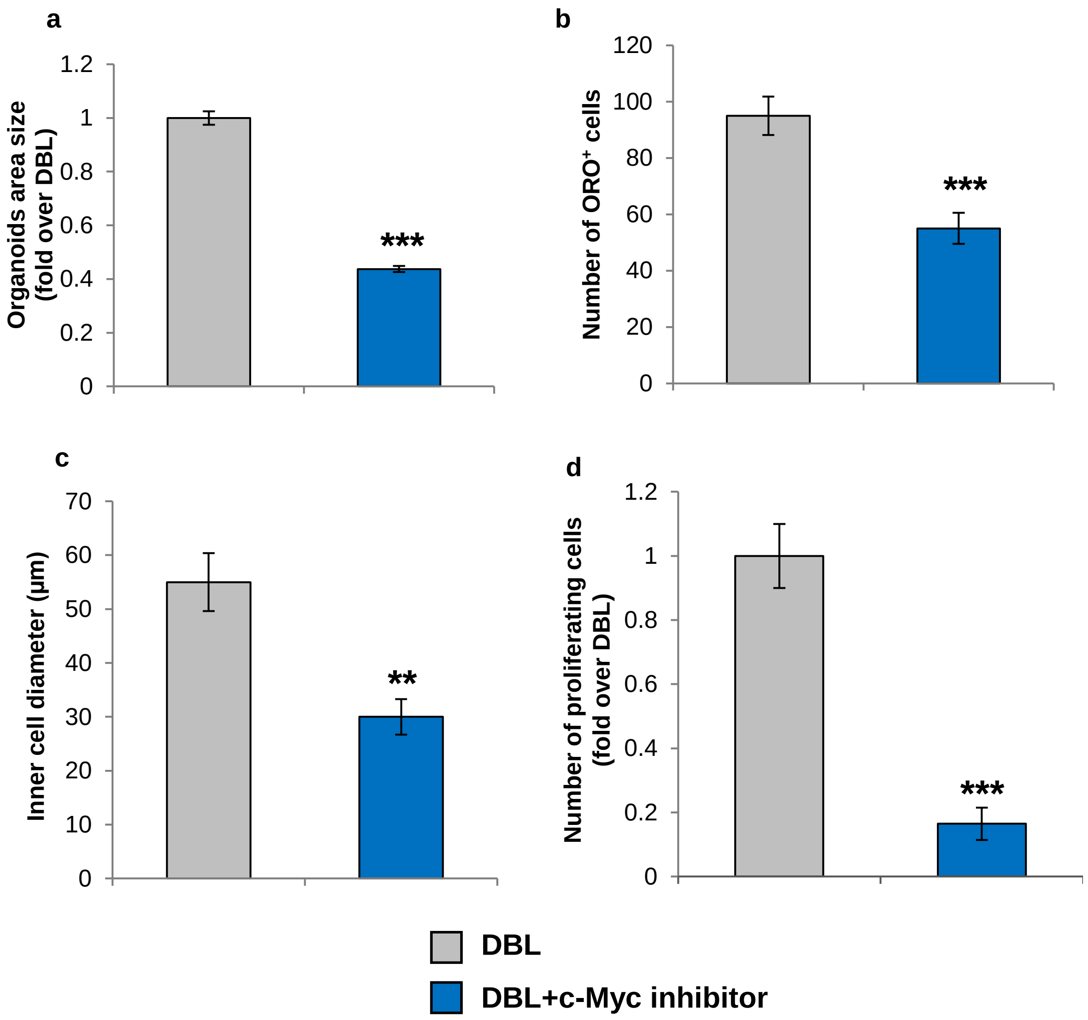

Supplementary Figure 8. c-Myc inhibitor serves as a potential treatment for acne vulgaris. (**a**-**d**) *Blimp1*-YFP^{+} cell-derived sebaceous gland organoids cultured for five days and treated for three days with Dihydrotestosterone, BRL-49653 and Linoleic acid combination (DBL), following treatments for three days with c-Myc inhibitor 10058-F4. (**a**) Size of treated organoids compared to control organoids. (**b**) Number of Oil red O (ORO)$^{+}$ cells in c-Myc inhibited organoids compared to DBL-treated organoids. (**c**) Analysis of treated and control organoids, measuring cell diameter of inner cells. (**d**) Number of Ki67^{+} proliferating cells in c-Myc inhibitor-treated organoids compared to DBL-treated organoids, showing significant reduction upon treatments with the inhibitor. Data are represented as the mean, error bars show ±s.e.m. [n = 120 organoids from 3 independent wells]. Significance was determined using unpaired, two-tailed Student's t test, where *p<0.05 **p<0.005, ***p<0.001.
