# Supplementary material for: Blimp1+ cells generate functional mouse sebaceous gland organoids in vitro
Source: Nat Commun. 2019 May 28;10:2348. doi: 10.1038/s41467-019-10261-6 (PMC6538623; doi:10.1038/s41467-019-10261-6)
Supplement: Supplementary file 4 — Reporting Summary [file 41467_2019_10261_MOESM4_ESM.pdf]

nature research

Corresponding author(s): Prof Yaron Fuchs

Last updated by author(s): Mar 25, 2019

# Reporting Summary

Nature Research wishes to improve the reproducibility of the work that we publish. This form provides structure for consistency and transparency in reporting. For further information on Nature Research policies, see Authors & Referees and the Editorial Policy Checklist.

## Statistics

For all statistical analyses, confirm that the following items are present in the figure legend, table legend, main text, or Methods section.

| n/a | Confirmed | |
|---|---|---|
| ☐ | ☒ | The exact sample size (*n*) for each experimental group/condition, given as a discrete number and unit of measurement |
| ☐ | ☒ | A statement on whether measurements were taken from distinct samples or whether the same sample was measured repeatedly |
| ☐ | ☒ | The statistical test(s) used AND whether they are one- or two-sided<br>*Only common tests should be described solely by name; describe more complex techniques in the Methods section.* |
| ☐ | ☒ | A description of all covariates tested |
| ☒ | ☐ | A description of any assumptions or corrections, such as tests of normality and adjustment for multiple comparisons |
| ☐ | ☒ | A full description of the statistical parameters including central tendency (e.g. means) or other basic estimates (e.g. regression coefficient) AND variation (e.g. standard deviation) or associated estimates of uncertainty (e.g. confidence intervals) |
| ☐ | ☒ | For null hypothesis testing, the test statistic (e.g. *F*, *t*, *r*) with confidence intervals, effect sizes, degrees of freedom and *P* value noted<br>*Give P values as exact values whenever suitable.* |
| ☒ | ☐ | For Bayesian analysis, information on the choice of priors and Markov chain Monte Carlo settings |
| ☒ | ☐ | For hierarchical and complex designs, identification of the appropriate level for tests and full reporting of outcomes |
| ☒ | ☐ | Estimates of effect sizes (e.g. Cohen's *d*, Pearson's *r*), indicating how they were calculated |

*Our web collection on statistics for biologists contains articles on many of the points above.*

## Software and code

Policy information about availability of computer code

| | |
|---|---|
| Data collection | Zen |
| Data analysis | Imaris, ImageJ, FSC Express Flow Cytometry Data Analysis, IDEAS®, Microsoft Excel and ZEN softwares. |

For manuscripts utilizing custom algorithms or software that are central to the research but not yet described in published literature, software must be made available to editors/reviewers. We strongly encourage code deposition in a community repository (e.g. GitHub). See the Nature Research guidelines for submitting code & software for further information.

## Data

Policy information about availability of data

All manuscripts must include a data availability statement. This statement should provide the following information, where applicable:

- Accession codes, unique identifiers, or web links for publicly available datasets
- A list of figures that have associated raw data
- A description of any restrictions on data availability

The authors declare that all data supporting the findings of this study are available within this article and its supplementary information files or from the corresponding author upon reasonable request.

# Field-specific reporting

Please select the one below that is the best fit for your research. If you are not sure, read the appropriate sections before making your selection.

☒ Life sciences ☐ Behavioural & social sciences ☐ Ecological, evolutionary & environmental sciences



October 2018

For a reference copy of the document with all sections, see nature.com/documents/nr-reporting-summary-flat.pdf

# Life sciences study design

All studies must disclose on these points even when the disclosure is negative.

| | |
|---|---|
| Sample size | At least three mice were analyzed in in vivo experiments. Organoid were grown from a mix of at least three mice and cultured in three independent biological repeats. |
| Data exclusions | No data was excluded from any analyses. |
| Replication | All experiments were repeated at least twice or three times as specified in the text. |
| Randomization | Mice were randomized and sample sizes included mixed sex. Age was matched (8 weeks). |
| Blinding | Investigators were blinded for statistical analyses of all microscopy quantifications. |

# Reporting for specific materials, systems and methods

We require information from authors about some types of materials, experimental systems and methods used in many studies. Here, indicate whether each material, system or method listed is relevant to your study. If you are not sure if a list item applies to your research, read the appropriate section before selecting a response.

## Materials & experimental systems

| n/a | Involved in the study | |
|---|---|---|
| ☐ | ☒ | Antibodies |
| ☒ | ☐ | Eukaryotic cell lines |
| ☒ | ☐ | Palaeontology |
| ☐ | ☒ | Animals and other organisms |
| ☒ | ☐ | Human research participants |
| ☒ | ☐ | Clinical data |

## Methods

| n/a | Involved in the study | |
|---|---|---|
| ☒ | ☐ | ChIP-seq |
| ☐ | ☒ | Flow cytometry |
| ☒ | ☐ | MRI-based neuroimaging |

## Antibodies

Antibodies used

Anti alpha human/mouse CD49f - (eBioscience, cat #12-0495-82, clone #eBioGoH3, lot #E01284-1632)
Anti Ly-6A/E - (BD Pharmigen, cat #558162, clone #D7, lot #5051949(
Anti Ki67 (eBioscience, cat #14-5698-80, clone #SolA15, lot #2002315)
Anti BrdU (Santa Cruz, cat #sc-32323, clone #IIB5, lot #F2016)
Anti keratin-5 (Abcam, cat #ab52635)
Anti keratin-15 (Abcam, cat #ab77832)
Anti MCM2 (Abcam, cat #ab4461)
Phalloidin (Life Technologies, cat #A22284, lot #1702518)

Validation

We rely on validation statements by manufacturers for all commercial antibodies.

## Animals and other organisms

Policy information about studies involving animals; ARRIVE guidelines recommended for reporting animal research

| | |
|---|---|
| Laboratory animals | Mus musculus - B6.Cg-Tg(Prdm1-EYFP)1Mnz/J mice. 8-week-old male and female mice were used randomly in this study. |
| Wild animals | *Provide details on animals observed in or captured in the field; report species, sex and age where possible. Describe how animals were caught and transported and what happened to captive animals after the study (if killed, explain why and describe method; if released, say where and when) OR state that the study did not involve wild animals.* |
| Field-collected samples | *For laboratory work with field-collected samples, describe all relevant parameters such as housing, maintenance, temperature, photoperiod and end-of-experiment protocol OR state that the study did not involve samples collected from the field.* |
| Ethics oversight | All animal studies received institutional ethics approval by the Pre-Clinical Research Authority (PCRA) of the Technion-Israel Institute of Technology and were performed in agreement with this authority's guidelines (ethics #IL0040115). |

Note that full information on the approval of the study protocol must also be provided in the manuscript.

## Flow Cytometry

### Plots

Confirm that:

☒ The axis labels state the marker and fluorochrome used (e.g. CD4-FITC).

☒ The axis scales are clearly visible. Include numbers along axes only for bottom left plot of group (a 'group' is an analysis of identical markers).

☒ All plots are contour plots with outliers or pseudocolor plots.

☒ A numerical value for number of cells or percentage (with statistics) is provided.

### Methodology

| | |
|---|---|
| Sample preparation | Dorsal skin from 8-week-old mouse was shaved and harvested. Underlying adipose tissue was removed before incubating in trypsin overnight at 4oC or 2 hr at 37oC. Epidermis and hairs were collected and filtered before sorting. |
| Instrument | BD FACSAria IIIu |
| Software | FSC Express Flow Cytometry Data Analysis |
| Cell population abundance | Cells were sorted by setting a predefined purity mask (16/32) on Blimp1-EYFP positive cells, as determined by histogram for fluorescence intensity (see below). Purity was examined by fluorescence microscopy post sorting (>95%). |
| Gating strategy | Unstained cell sample was obtained by using littermates negative for Blimp1-EYFP. This sample was used to set PMT voltages, perform compensation and gate FSC-A/SSC-A. Dead cells and debris were gated out according to FSC and SSC properties and DAPI signal. Out of this parent population singlets were gated using FSC-W vs. FSC-H. Sorted cells were gated out of the singlets according to Integrin alpha six positivity and from this parent population Sca1 negativity and EYFP positivity (Fig. 1b). |

☐ Tick this box to confirm that a figure exemplifying the gating strategy is provided in the Supplementary Information.
