# Supplementary material for: Blimp1+ cells generate functional mouse sebaceous gland organoids in vitro
Source: Nat Commun. 2019 May 28;10:2348. doi: 10.1038/s41467-019-10261-6 (PMC6538623; doi:10.1038/s41467-019-10261-6)
Supplement: Supplementary file 5 — Description of Additional Supplementary Files [file 41467_2019_10261_MOESM5_ESM.docx]

**Title:** Supplementary Movie 1.
**Description:** Continuous Z-stack projections of sebaceous gland organoid stained with phalloidin and DAPI. Note the presence of smaller sebocytes on the outer rim and larger sebocytes towards the inner zone.

**Title:** Supplementary Movie 2.
**Description:** Live imaging utilizing light sheet fluorescence microscopy of movement kinetics in sebaceous gland organoids. 7-day old organoid derived from Blimp1-YFP-H2B-GFP+ (nuclear labeled) cells are captured over a 24 hour period. Two tracked cells and their progeny (blue and red dots) over time are marked.
